# Catalytic isoforms of AMP-activated protein kinase differentially regulate IMPDH activity and photoreceptor neuron function

**DOI:** 10.1172/jci.insight.173707

**Published:** 2024-01-16

**Authors:** Tae Jun Lee, Yo Sasaki, Philip A. Ruzycki, Norimitsu Ban, Joseph B. Lin, Hung-Ting Wu, Andrea Santeford, Rajendra S. Apte

**Affiliations:** 1 John F. Hardesty, MD Department of Ophthalmology and Visual Sciences;; 2 Department of Developmental Biology; and; 3 Department of Genetics, Washington University in St. Louis School of Medicine, St. Louis, Missouri, USA.; 4 Department of Ophthalmology, Keio University School of Medicine, Tokyo, Japan.; 5 Department of Medicine, Washington University in St. Louis School of Medicine, St. Louis, Missouri, USA.

**Keywords:** Metabolism, Ophthalmology, Bioenergetics, Protein kinases, Signal transduction

## Abstract

AMP-activated protein kinase (AMPK) plays a crucial role in maintaining ATP homeostasis in photoreceptor neurons. AMPK is a heterotrimeric protein consisting of α, β, and γ subunits. The independent functions of the 2 isoforms of the catalytic α subunit, PRKAA1 and PRKAA2, are uncharacterized in specialized neurons, such as photoreceptors. Here, we demonstrate in mice that rod photoreceptors lacking PRKAA2, but not PRKAA1, showed altered levels of cGMP, GTP, and ATP, suggesting isoform-specific regulation of photoreceptor metabolism. Furthermore, PRKAA2-deficient mice displayed visual functional deficits on electroretinography and photoreceptor outer segment structural abnormalities on transmission electron microscopy consistent with neuronal dysfunction, but not neurodegeneration. Phosphoproteomics identified inosine monophosphate dehydrogenase (IMPDH) as a molecular driver of PRKAA2-specific photoreceptor dysfunction, and inhibition of IMPDH improved visual function in *Prkaa2* rod photoreceptor–knockout mice. These findings highlight a therapeutically targetable PRKAA2 isoform–specific function of AMPK in regulating photoreceptor metabolism and function through a potentially previously uncharacterized mechanism affecting IMPDH activity.

## Abstract 2

<p>Rod photoreceptor-specific deletion of the AMPK catalytic isoform PRKAA2 results in inosine monophosphate dehydrogenase&nbsp;hyperactivity, which leads to visual function deficits that can be&nbsp;improved with mycophenolate.</p>

## Graphical abstract

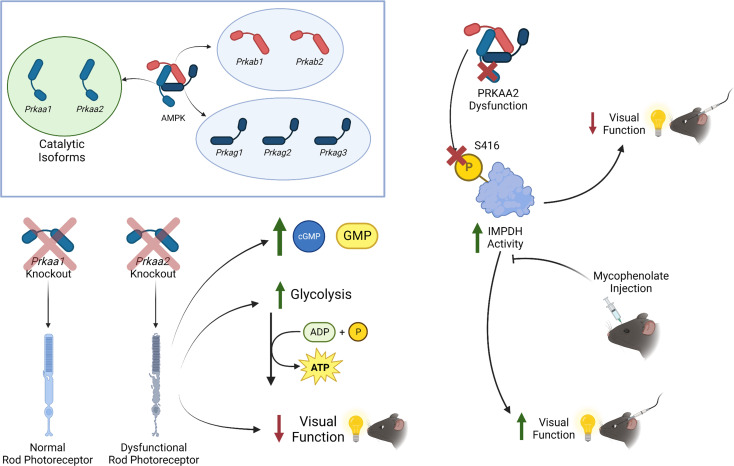

## Introduction

AMP-activated protein kinase (AMPK) is a crucial regulator of energy homeostasis and is considered a central player in replenishing low adenosine triphosphate (ATP) levels by initiating catabolic processes (1). Prior studies using both gain- and loss-of-function approaches have investigated the role of AMPK in maintaining the high energy demands of the neurosensory retina in homeostasis and during aging, light-induced oxidative stress, and inflammation (2–4). AMPK has broadly been reported in multiple animal and tissue models to regulate mitochondrial biogenesis, autophagy, glycolysis and gluconeogenesis, β-oxidation and lipogenesis, and protein metabolism (5–9). As a trimeric protein, AMPK has different isoforms for its 3 subunits (α, β, γ) with gene names beginning with “PRKA,” which result in 12 different possible combinations to comprise the complete heterotrimeric enzyme (10). The α subunit, the catalytic subunit, has 2 isoforms in mammals (PRKAA1 and PRKAA2, also called AMPKα1 and AMPKα2, respectively) and is responsible for regulating targets via phosphorylation. The β subunit, a regulatory subunit, has 2 isoforms in mammals (PRKAB1 and PRKAB2, also called AMPKβ1 and AMPKβ2, respectively) and is necessary for allosteric activation of the α subunit. The γ subunit, a regulatory subunit, has 3 isoforms in mammals (PRKAG1, PRKAG2, and PRKAG3, also called AMPKγ1, AMPKγ2, and AMPKγ3, respectively) and binds to AMP to initiate allosteric activation of the α subunit. Previous investigations have suggested that PRKAA1 and PRKAA2 are not fully redundant through generation and phenotypic characterization of *Prkaa1*- and *Prkaa2*-null mutant mice, demonstrating profound effects and embryonic lethality in the case of double *Prkaa1* and *Prkaa2* knockout (11). Other studies have examined the effects of catalytic isoform–specific null mutant phenotypes, suggesting isoform-specific roles in specific tissues and contexts (12).

However, the specific mechanistic contributions of the individual catalytic isoforms of AMPK in supporting retinal function have not been elucidated. Although some compounds such as AMP analog C2 target a specific catalytic isoform (13), most existing pharmaceuticals that target AMPK, such as the widely prescribed type 2 diabetes drug metformin, do so nonspecifically (14–16); they do not specifically target the catalytic subunit. As such, which isoform contributes to therapeutic effects is still unknown. Understanding the potentially unique roles of the catalytic isoforms of AMPK is thus critical to provide molecular mechanistic insight into the pharmacologic effects of AMPK agonists.

A pressing challenge in understanding the molecular roles of AMPK is bridging the heterogeneity and diversity of AMPK targets across different tissue and cell types, especially considering the many heterotrimeric flavors AMPK presents as across tissues (17). However, discerning the functions of PRKAA1 and PRKAA2 within each tissue is difficult because of biased expression profiles across different tissues, so a molecular investigation to better understand the potentially unique roles of PRKAA1 and PRKAA2 is warranted given the significance of AMPK in regulating energy homeostasis. For example, murine leukocytes predominantly express one of the AMPK catalytic isoforms (*Prkaa1*) over the other, making it difficult to investigate the role of individual catalytic isoforms in these cells (18). However, transcriptomic studies of the neurosensory retina have shown appreciable expression of both isoforms, offering the opportunity to investigate the functions of both catalytic isoforms in an energy-demanding, complex neurovascular tissue (19). We hypothesized that the 2 catalytic isoforms display distinct metabolic roles in regulating photoreceptor function in the retina.

The neurosensory retina is composed of energy-demanding neurons, with roughly 80% of the cell population being rod photoreceptor neurons (20). Photoreceptors are highly metabolically active; they are essential for vision and especially vulnerable to metabolic perturbations (21). Previous studies have demonstrated that mutations in genes involved in energy homeostasis, the visual cycle, or the phototransduction cascade typically result in photoreceptor dysfunction and loss (22–26). Specialized features of rod photoreceptors predispose them to metabolic vulnerabilities. Known as the Warburg effect (27), photoreceptors heavily rely on aerobic glycolysis to meet the energy demands of both dark current maintenance as well as anabolic generation of new outer segments (21, 22). The dark current is the depolarizing current necessary to appropriately respond to light stimuli while the rod photoreceptor is unstimulated. Although nonproliferative, rod photoreceptors have high anabolic demand due to their diurnal shedding and regeneration of outer segments: specialized organelles rich with membranes holding proteins necessary for phototransduction. Both of these features are unique to rod photoreceptors and are highly dependent on aerobic glycolysis. As such, it is important to investigate the catalytic functions of AMPK that regulate photoreceptor metabolism, as impaired AMPK activity leads to photoreceptor dysfunction and subsequent vision loss.

In addition to high ATP demand, photoreceptors require a delicate balance of other metabolites, such as guanosine monophosphate (GMP) and cyclic guanosine monophosphate (cGMP) for normal phototransduction (28). This is due to their unique reliance on cGMP-gated membrane channels and guanosine triphosphate–dependent (GTP-dependent) transducin activation in rod photoreceptor outer segments. The phototransduction cascade is initiated with a light photon activating an opsin protein, eventually leading to active conversion of cGMP to GMP by phosphodiesterase. The resulting decrease in intracellular cGMP concentration forces cGMP-gated channels to be closed, subsequently hyperpolarizing the membrane to initiate the visual transduction cascade to downstream effectors. Reliance on both ATP and GTP homeostasis for critical functions predisposes rod photoreceptors to unique vulnerabilities when phospho-purine homeostasis is impaired. As the de novo purine synthesis pathway intricately connects ATP and GTP homeostasis (29), elucidating the functions of AMPK in rod photoreceptors may offer molecular insights into photoreceptor metabolism and function.

Here, we establish that the AMPK catalytic isoforms PRKAA1 and PRKAA2 have distinct functions in rod photoreceptors and identify a molecular basis of energy regulation and photoreceptor dysfunction in the setting of selective PRKAA2 dysfunction. Abrogation of PRKAA2, but not PRKAA1, function in rod photoreceptors leads to structural abnormalities and visual dysfunction. Furthermore, metabolomic and phosphoproteomic studies reveal PRKAA2 dysfunction causes aberrant inosine monophosphate dehydrogenase (IMPDH) activity, leading to rod photoreceptor dysfunction. We also demonstrate visual function deficits caused by PRKAA2 dysfunction can be improved with an IMPDH antagonist, a finding that has therapeutic possibilities. Taken together, this study mechanistically delineates the functions of PRKAA1 and PRKAA2 in rod photoreceptor neurons and establishes IMPDH as a target of PRKAA2.

## Results

### Prkaa1 and Prkaa2 expression in the retina.

**Figure 1:**
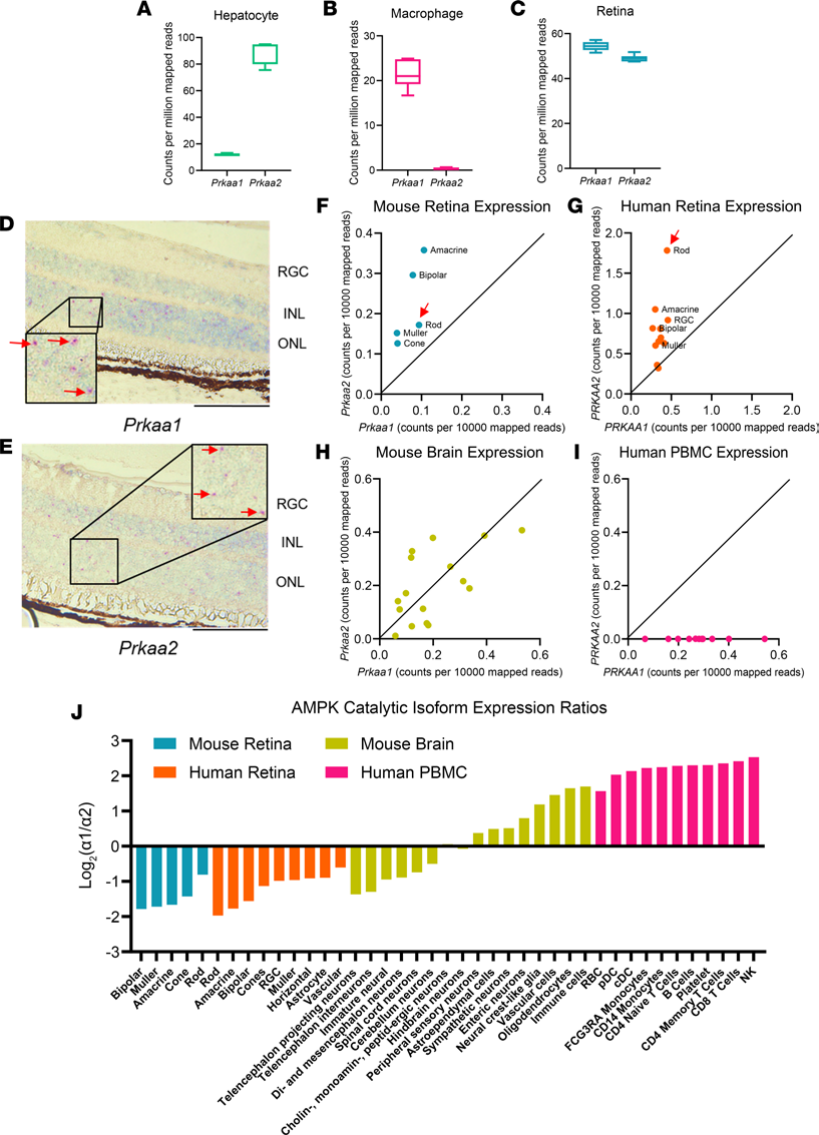
Both catalytic isoforms of AMPK are appreciably expressed in the retina with consistent expression profiles across human and mouse retinas. (**A**–**C**) Gene expression of *Prkaa1* and *Prkaa2* (counts per million mapped reads) across different mouse tissues. Box plots show interquartile range, median (line), and minimum and maximum (whiskers). (**A**) Hepatocytes (*n* = 4) of mice favor *Prkaa2* over *Prkaa1* expression while (**B**) macrophages (*n* = 8) of mice favor *Prkaa1* over *Prkaa2* expression. (**C**) Mouse retinas demonstrate appreciable expression levels of *Prkaa1* and *Prkaa2* (*n* = 6). (**D** and **E**) In situ hybridization of wild-type retina sections confirmed expression of (**D**) *Prkaa1* and (**E**) *Prkaa2*, seen in magenta dots within the outer nuclear layer (red arrows), suggesting expression of both isoforms in rod photoreceptors. RGC, retinal ganglion cells. (**F**) Scatterplot of *Prkaa1* and *Prkaa2* expression profiles of mouse retina cell types from single-cell RNA-sequencing data. The cell types show appreciable *Prkaa1* and *Prkaa2* expression. The rod photoreceptor cluster (red arrow) shows roughly a 2-fold expression of *Prkaa2* over *Prkaa1*. (**G**) Scatterplot of PRKAA1 and PRKAA2 expression profiles of human retina cell types. The cell types show appreciable PRKAA1 and PRKAA2 expression. The rod photoreceptor cluster (red arrow) shows roughly a 3-fold expression of *Prkaa2* over *Prkaa1*. (**H**) Scatterplot of *Prkaa1* and *Prkaa2* expression profiles of mouse brain cell types. Most cell types in the mouse brain demonstrate appreciable expression of *Prkaa1* and *Prkaa2*. (**I**) Scatterplot of PRKAA1 and PRKAA2 expression profiles of human PBMC types. These cell types do not appreciably express PRKAA2, unlike the central nervous system tissues. (**J**) Waterfall graph depicting the expression ratios of both catalytic isoforms across mouse retina, human retina, mouse brain, and human PBMCs. The majority of neuronal cell types express the α2 isoform over α1 whereas immune cells overwhelmingly express the α1 isoform over α2. Scale bar: 100 μm; insets: 20× original magnification.

Delineating the independent functions of PRKAA1 and PRKAA2 in vivo remains challenging because of the biased expression profiles of the catalytic isoforms across different tissue types where the expression of only 1 isoform often dominates. Using publicly available bulk RNA-sequencing data (19), we compared the expression levels of both isoforms in mouse retina and across mouse hepatocytes and macrophages through publicly available bulk RNA-sequencing data (Figure 1, A–C) (18, 30). While the retina appreciably expresses both isoforms, hepatocytes and macrophages demonstrate markedly skewed expression profiles, with dominant expression of only 1 of the catalytic isoforms. As approximately 80% of the mouse retina is comprised of rod photoreceptors that are highly metabolically active, we sought to investigate the functions of PRKAA1 and PRKAA2 in rod photoreceptors (20). Using in situ hybridization, we were able to visualize the expression of both *Prkaa1* and *Prkaa2* in the outer nuclear layer of retina sections of wild-type mice, which verified that both isoforms were expressed in rod photoreceptors (Figure 1, D and E).

### Expression profiles of AMPK catalytic isoforms are consistent across mouse and human retinas.

We further sought to assess if we could appreciate comparable expression profiles of both isoforms between mouse and human retinas. To investigate this, we utilized publicly available single-cell RNA-sequencing data sets of both mouse and human retinas (31, 32). We annotated clusters based on known marker genes, then calculated average normalized expression of *PRKAA1* and *PRKAA2* within each cell type (Figure 1, F and G). Both mouse and human data indicated that retina cells expressed both isoforms above background levels but suggested some preference for the α2 isoform. Interestingly, a similar pattern of coexpression of both isoforms was also observed across a variety of cell types within the mouse brain (33) (Figure 1H), but was in stark contrast with expression within individual human PBMC types, which demonstrated near-complete restriction to *PRKAA1* expression (Figure 1I). Taken together, these data justify the study of isoform-specific roles in central nervous system tissues in mice and present rod photoreceptors as an excellent model.

We also plotted the relative expression of α1 and α2 isoforms within each cell type. Again, this quantitative assessment displayed a preference for the α2 isoform within all mouse and human retinal cell types (Figure 1J). Mouse brain data suggested a split in expression preference between cell types, but closer observation showed more neuronal cell types preferred *Prkaa2* expression while immune cells and glia expressed more *Prkaa1*. Human PBMC data demonstrated that these cells overwhelmingly expressed *PRKAA1*. Taken together, these data supported the notion that neuronal cell types express both isoforms but with some preference for α2. Non-neuronal and immune cells showed a strong preference for α1, with little to no appreciable expression of α2. Given the consistent isoform expression profiles across neuronal cell types, including retinal rod photoreceptor neurons, rod cells provide an accessible and important cell type to investigate the functions of the individual isoforms that may have functional implications for other neurons in the central nervous system.

### Prkaa1^-Rhod/-Rhod^ and Prkaa2^-Rhod/-Rhod^ express deficient PRKAA1 and PRKAA2, respectively, in rod photoreceptors.

**Figure 2:**
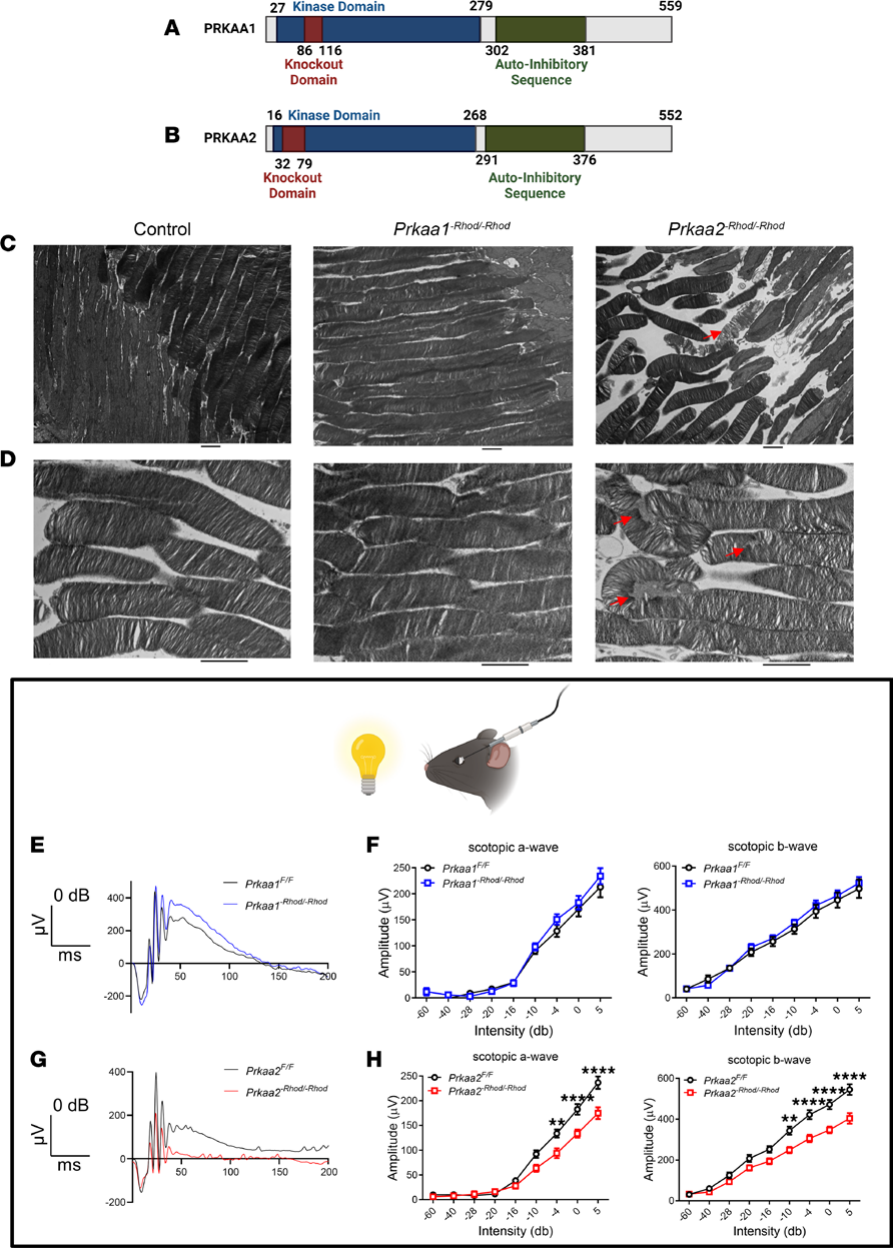
PRKAA2, but not PRKAA1, dysfunction leads to rod photoreceptor structural abnormalities and visual dysfunction. (**A** and **B**) Schematic representations of the protein sequences in the *Prkaa1^-Rhod/-Rhod^* and *Prkaa2^-Rhod/-Rhod^* models, respectively. Knockout domains are within the kinase domain to abrogate function but preserve overall expression of the proteins. (**A**) Schematic representation of the PRKAA1 and (**B**) PRKAA2 protein sequence. (**C**) Representative transmission electron microscopy images of magnification, 2,500×, of rod photoreceptors. (Left) Rod photoreceptors of control mice (*Prkaa2^fl/fl^*) demonstrate similar features to those of *Prkaa1^-Rhod/-Rhod^* with consistent membrane structure and organization. (Middle) Rod photoreceptors of *Prkaa1^-Rhod/-Rhod^* demonstrate intact connections between the outer and inner segments with consistent laminar organization of the outer segment membranes. (Right) Rod photoreceptors of *Prkaa2^-Rhod/-Rhod^* consistently demonstrate detachments between the outer and inner segments and occasional outer segment membrane dysmorphisms (red arrow). (**D**) Representative transmission electron microscopy images of magnification, 6,000×, of the outer segments of rod photoreceptors. (Left) Outer segments from control mice (*Prkaa2^fl/fl^*) show consistent striations and laminar organization resembling *Prkaa1^-Rhod/-Rhod^*. (Middle) *Prkaa1^-Rhod/-Rhod^* outer segments demonstrated organized and laminar structure indicative of normal wild-type structure. (Right) *Prkaa2^-Rhod/-Rhod^* outer segments exhibited disorganized membrane layers and occasional manifestation of granular debris (red arrows). (**E** and **F**) Scotopic electroretinography of *Prkaa1^-Rhod/-Rhod^* and respective wild-type littermates. (**E**) Representative traces of 0 dB intensity flashes demonstrate similar waveforms between *Prkaa1^fl/fl^* and *Prkaa1^-Rhod/-Rhod^* (*n* = 7). (**F**) Analyses of scotopic a (left) and scotopic b (right) amplitude measurements reveal no significant changes in *Prkaa1^-Rhod/-Rhod^*. (**G** and **H**) Scotopic electroretinography of *Prkaa2^-Rhod/-Rhod^* and respective wild-type littermates (*n* = 7). (**G**) Representative traces of 0 dB intensity flashes show a diminutive waveform from *Prkaa2^-Rhod/-Rhod^*. (**H**) Analyses of scotopic a (left) and scotopic b (right) amplitude measurements confirm significant attenuation in *Prkaa2^-Rhod/-Rhod^* measurements (**P < 0.01, ****P < 0.0001 by 2-way ANOVA with post hoc Bonferroni’s multiple comparisons test). Values are mean ± SEM. Scale bars represent 2 μm. Representative images selected from 40 images for each group.

We generated *Prkaa1* rod photoreceptor–specific knockouts (*Prkaa1^-Rhod/-Rhod^*) with wild-type littermates (*Prkaa1^fl/fl^*) and *Prkaa2* rod photoreceptor–specific knockouts (*Prkaa2^-Rhod/-Rhod^*) with wild-type littermates (*Prkaa2^fl/fl^*) by crossing floxed mice with mice carrying 1 copy of the rhodopsin-iCre75 transgene. We performed quantitative revers transcription PCR (qRT-PCR) of magnetically isolated rod photoreceptors to verify both knockout of the targeted region and lack of any compensatory overexpression of the other catalytic isoform (Supplemental Figure 1; supplemental material available online with this article; https://doi.org/10.1172/jci.insight.173707DS1) (26). Despite mild decrease in expression of *Prkaa2* in *Prkaa1^-Rhod/-Rhod^*, we did not observe structural or functional deficits in *Prkaa1^-Rhod/-Rhod^* rod photoreceptors (Figure 2, C–F).

Both *Prkaa1^fl/fl^* and *Prkaa2^fl/fl^* mice have been previously characterized (34). Both protein sequences are highly homologous, with kinase domains and auto-inhibitory sequences of similar size and location (Figure 2, A and B). The knockout domains were designed to abrogate kinase function but preserve overall protein expression. We hypothesized that PRKAA1 and PRKAA2 carry distinct roles in maintaining rod photoreceptor structure and function.

### Prkaa2^-Rhod/-Rhod^ rod photoreceptors exhibit disorganized outer segment structures on electron microscopy.

To structurally characterize the photoreceptors of both *Prkaa1^-Rhod/-Rhod^* and *Prkaa2^-Rhod/-Rhod^* mice, we used histological techniques. We enucleated eyes from both *Prkaa1^-Rhod/-Rhod^* and *Prkaa2^-Rhod/-Rhod^* mice and processed sagittal sections of the retina and stained with hematoxylin and eosin (H&E). Neither *Prkaa1^-Rhod/-Rhod^* nor *Prkaa2^-Rhod/-Rhod^* retina sections exhibited gross anatomical abnormalities in the outer nuclear layer, outer segment, and inner segment layers where rod photoreceptors reside (Supplemental Figure 2). We next evaluated ultrastructural changes that are unappreciable using H&E staining techniques. Using transmission electron microscopy, we were able to consistently observe outer segment changes only in *Prkaa2^-Rhod/-Rhod^* photoreceptors (Figure 2, C and D). The structural integrity of the outer segments was visibly compromised, with accumulation of granular debris and loss of laminar organization. *Prkaa1^-Rhod/-Rhod^* outer segments appeared unchanged compared to controls. These data taken together suggest *Prkaa2^-Rhod/-Rhod^* rod photoreceptors have altered outer segment structural integrity without overt degeneration.

Outer segments of rod photoreceptors are specialized organelles responsible for responding to light photons by converting them into electrical signals (22). These organelles are responsible for the bulk of ATP demand from the cell, as maintaining membrane electrochemical sensitivity is ATP intensive. We therefore hypothesized that the lack of outer segment structural integrity in *Prkaa2^-Rhod/-Rhod^* rod photoreceptors may lead to altered visual function.

### Prkaa2^-Rhod/-Rhod^ mice demonstrate visual function deficits in vivo.

We utilized in vivo full-field scotopic electroretinography to assess rod photoreceptor function (35) in both *Prkaa1^-Rhod/-Rhod^* and *Prkaa2^-Rhod/-Rhod^* mice. Dark-adapted mice were anesthetized and retinal responses to increasing intensity levels of white light flashes were recorded. Initial hyperpolarized deflections from the baseline were characterized as the scotopic a wave, the putative measurement of rod photoreceptor response, while the trough of the a wave to the crest of the deflection were characterized as the scotopic b wave, the putative measurement of the inner retinal response from rod photoreceptor hyperpolarization. Consistent with ultrastructural photoreceptor outer segment changes, we observed significantly attenuated scotopic a and scotopic b waves in *Prkaa2^-Rhod/-Rhod^* (Figure 2, G and H), but not in *Prkaa1*^-Rhod/-Rhod^ (Figure 2, E and F), suggesting *Prkaa2^-Rhod/-Rhod^* rod photoreceptors were functionally impaired. These data support that PRKAA2 is the key catalytic AMPK isoform in rod photoreceptors.

### cGMP, GTP, and ATP levels are elevated in Prkaa2^-Rhod/-Rhod^ retinas.

**Figure 3:**
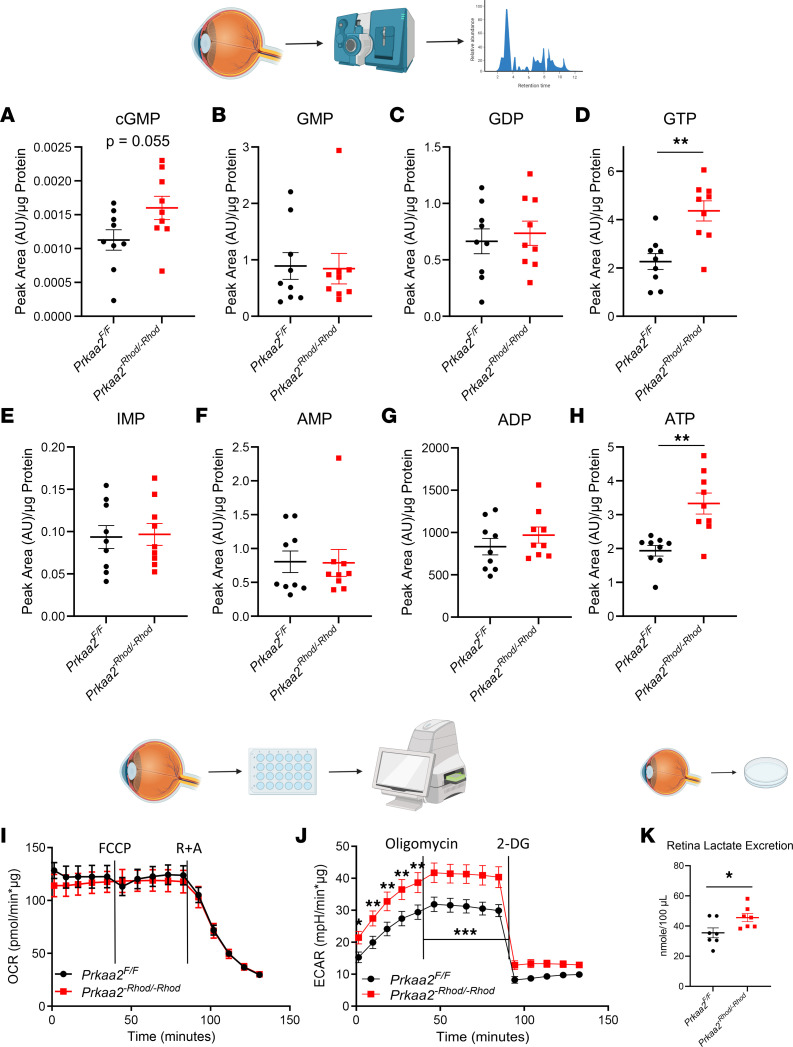
*Prkaa2^-Rhod/-Rhod^* retinas demonstrate phospho-purine overproduction and increased glycolysis. (**A**–**H**) Metabolomics analyses of different phospho-purines in *Prkaa2^-Rhod/-Rhod^* retinas using LC-MS/MS (*n* = 9). (**A**) *Prkaa2^-Rhod/-Rhod^* retinas demonstrated near-significant increase in cGMP levels, a critical regulator of the dark current (*P* = 0.056 by Welch’s *t* test). (**B** and **C**) GMP and GDP levels were not significantly changed in *Prkaa2^-Rhod/-Rhod^*. (**D**) GTP levels were significantly increased in *Prkaa2^-Rhod/-Rhod^* (**P < 0.01 by Welch’s *t* test). (**E**) Levels of IMP, a precursor to GMP, were not significantly changed in *Prkaa2^-Rhod/-Rhod^*. (**F** and **G**) AMP and ADP levels were not significantly changed in *Prkaa2^-Rhod/-Rhod^*. (**H**) ATP levels were significantly increased in *Prkaa2^-Rhod/-Rhod^* (**P < 0.01 by Welch’s *t* test). (**I** and **J**) Extracellular flux analyses by retina Seahorse of *Prkaa2^-Rhod/-Rhod^*. (**I**) Oxygen consumption rate as a measure of oxidative phosphorylative flux was not significantly changed in *Prkaa2*^-Rhod/-Rhod^ (*n* = 7). (**J**) Extracellular acidification rate as a measure of glycolytic flux was significantly increased (*P* < 0.0001 by 2-way ANOVA, comparing the wild-type group to the knockout group as a whole). Glycolysis (*P < 0.05, **P < 0.01 by post hoc Bonferroni’s multiple comparisons test) and glycolytic capacity (***P < 0.001 by post hoc Bonferroni’s multiple comparisons test) were significantly upregulated in *Prkaa2^-Rhod/-Rhod^* (*n* = 8). (**K**) Excreted retina lactate levels were significantly increased in *Prkaa2^-Rhod/-Rhod^*, supporting the increased glycolysis phenotype (*n* = 7, *P < 0.05 by Welch’s *t* test). Values are mean ± SEM.

As AMPK is known to modulate the metabolome and cellular energetics (1), we next determined whether differences in phospho-purines, such as ATP and GTP, might explain the structural and functional deficits observed in the *Prkaa2^-Rhod/-Rhod^* mice. To investigate this, we utilized whole-retina liquid chromatography-tandem mass spectrometry–based (LC-MS/MS–based) metabolomics to quantify different metabolite levels. AMP, ADP, GMP, GDP, and IMP levels were not significantly changed in *Prkaa2^-Rhod/-Rhod^* retinas (Figure 3, B, C, and E–G). Surprisingly, we discovered significantly increased levels of ATP (1.723-fold) and GTP (1.927-fold) in *Prkaa2^-Rhod/-Rhod^* retinas (Figure 3, D and H). In addition, cGMP showed an increasing trend (1.42-fold, *P* = 0.055) (Figure 3A). These metabolites were unchanged in *Prkaa1^-Rhod/-Rhod^* retinas (Supplemental Figure 3). Increased levels of cGMP and GTP provide insight into potential mechanisms of rod photoreceptor dysfunction, as cGMP and GTP homeostasis is critical in the phototransduction cascade and rod dark current maintenance (21, 28). Although AMPK has widely been reported to help modulate catabolic processes contributing to ATP production, these data suggest PRKAA1 or PRKAA2 dysfunction did not negatively impact the steady-state pool of ATP in rod photoreceptors. On the contrary, PRKAA2 dysfunction was associated with increased steady-state ATP levels. These changes could reflect diminished energy consumption. Increased ATP levels can also be driven by de novo AMP synthesis and the ATP salvage pathway through glycolysis, tricarboxylic acid cycle, and the electron transport chain, so we next sought to elucidate the mechanism behind increased ATP production (7, 22, 29).

### Prkaa2^-Rhod/-Rhod^ retinas demonstrate increased glycolytic flux.

Rod photoreceptors primarily use aerobic glycolysis for ATP production to meet their high energy demand (27). To elucidate the metabolic mechanism underlying the increased ATP in *Prkaa2^-Rhod/-Rhod^* retinas, we performed extracellular flux analyses of retinas to assess both oxidative phosphorylation and glycolytic flux capacity using the Seahorse MitoStress and Glycolytic Stress test kits, respectively (36). We did not observe any changes in oxidative phosphorylative flux (Figure 3I); however, we observed a significant increase in glycolytic flux in *Prkaa2^-Rhod/-Rhod^* (Figure 3J). We did not observe significant changes to extracellular flux capacity in *Prkaa1^-Rhod/-Rhod^* retinas (Supplemental Figure 4, A and B). To further corroborate our findings, we extracted retinas and cultured them in Ames’ Media for 30 minutes in a 5% CO_2_ cell culture incubator to measure excreted lactate levels in the supernatant. We used a colorimetric plate-based assay to measure lactate levels. As lactate is a by-product of glycolysis and has been reported to be excreted by rod photoreceptors (37), we anticipated an increase of lactate excretion in *Prkaa2^-Rhod/-Rhod^* retinas. Indeed, we observed significantly increased levels of excreted lactate in *Prkaa2^-Rhod/-Rhod^* (Figure 3K) but not *Prkaa1^-Rhod/-Rhod^* retinas (Supplemental Figure 4C). These data support that *Prkaa2^-Rhod/-Rhod^* rod photoreceptors demonstrate increased glycolysis whereas *Prkaa1^-Rhod/-Rhod^* rods do not. Moreover, these data also suggest that the mechanism behind increased ATP levels in *Prkaa2^-Rhod/-Rhod^* is activated glycolysis.

### Rod-isolated phosphoproteomics reveals IMPDH as a downstream effector of PRKAA2.

**Figure 4:**
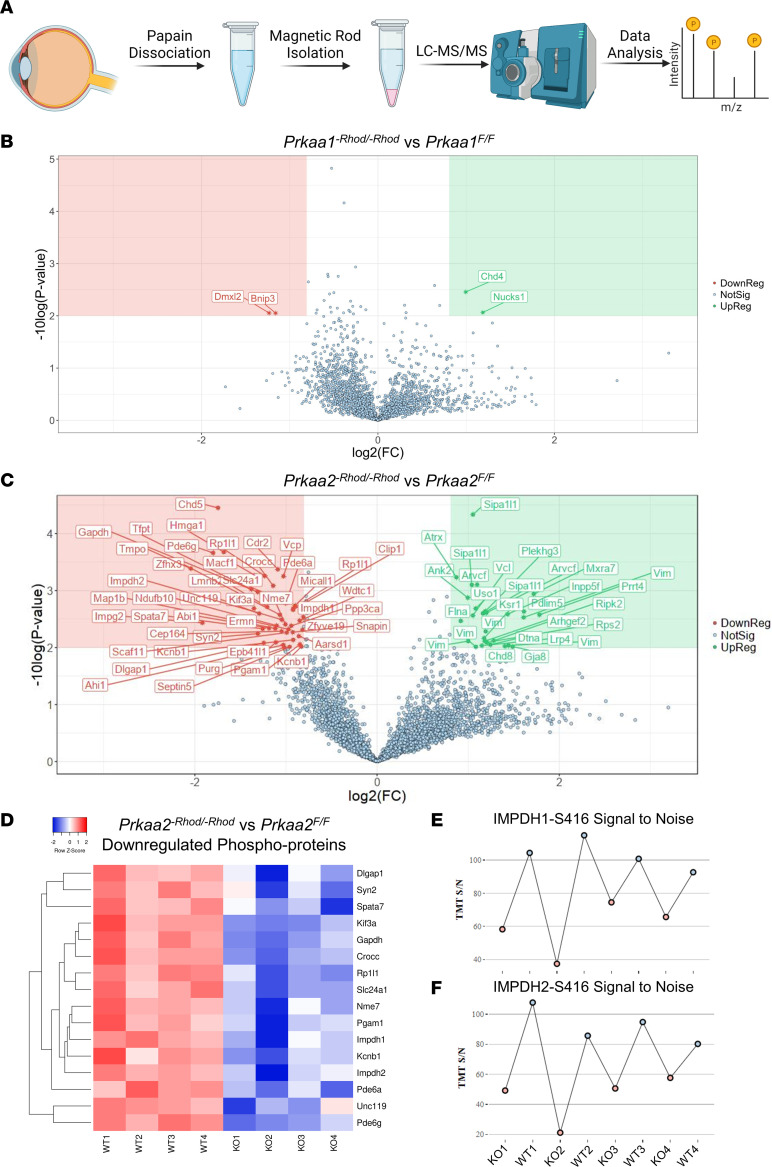
PRKAA2 dysfunction leads to wide changes to the phosphoproteome and decreased S416 phosphorylation of IMPDH. (**A**) Schematic representation of the workflow to isolate rod photoreceptors to use for phosphoproteomics analysis. Six retinas were dissected per sample and first dissociated using papain digestion. CD73-PE and Rhodopsin-biotin antibodies were used to tag rod photoreceptor inner and outer segments and were isolated using immunomagnetic beads. The cells were then lysed and processed by LC-MS/MS using an unbiased phosphoproteomics pipeline and analyzed. (**B** and **C**) Rod photoreceptors from *Prkaa1^-Rhod/-Rhod^* and *Prkaa2^-Rhod/-Rhod^* were processed for phosphoproteomic analyses (*n* = 4). 1.75 fold-change and 0.01 *P* value cutoffs with < 1% false discovery rate were used to determine significant changes. (**B**) *Prkaa1^-Rhod/-Rhod^* analysis revealed 2 downregulated targets, which were unrelated to rod photoreceptor function. (**C**) *Prkaa2^-Rhod/-Rhod^* analysis revealed 45 downregulated targets, including species related to the phototransduction cascade and photoreceptor function. (**D**) A selection of downregulated phosphoproteins from the *Prkaa2^-Rhod/-Rhod^* phosphoproteomics data set were plotted on a heatmap to visualize the spread of individual samples. Samples with higher *z* scores are visualized as a deeper red color while samples with lower *z* scores are visualized as a deeper blue color. Each row represents a phospho-site of the denoted protein, while each column represents either a sample from *Prkaa2^fl/fl^* (WT) or *Prkaa2^-Rhod/-Rhod^* (KO). (**E** and **F**) Tandem mass tag (TMT) signal-to-noise ratios of IMPDH1-S416 and IMPDH2-S416 from individual *Prkaa2^fl/fl^* (WT) and *Prkaa2^-Rhod/-Rhod^* (KO) samples. KO samples are colored as pink dots while WT samples are colored as blue dots. In both IMPDH1-S416 and IMPDH2-S416 measurements, the KO samples overall present lower signal-to-noise ratios than WT samples.

We next sought to identify candidate targets of PRKAA2 that mediate the structural and functional phenotype of *Prkaa2^-Rhod/-Rhod^*, particularly the mechanism behind the increased cGMP and GTP levels observed through metabolomics. As AMPK phosphorylates downstream targets through its enzymatic activity, we utilized an immuno-magnetic precipitation to isolate rod photoreceptors for unbiased LC/MS-MS phosphoproteomics from both *Prkaa1^-Rhod/-Rhod^* and *Prkaa2^-Rhod/-Rhod^* (Figure 4A). Phosphoproteomics of *Prkaa1^-Rhod/-Rhod^* rod photoreceptors revealed relatively minor changes to the phosphoproteome unrelated to metabolic changes (Figure 4B). However, phosphoproteomics of *Prkaa2^-Rhod/-Rhod^* revealed a vastly altered phosphoproteome compared with wild-type controls (Figure 4C).

A total of 4,086 unique phospho-sites were quantified, and 73 phospho-sites were found to be significantly changed with < 1% false discovery rate with 1.75 fold-change and 0.01 *P* value cutoffs. Consistent with diminished visual function on electroretinography, numerous proteins involving the phototransduction cascade and photoreceptor function demonstrated differential phosphorylation, including retinitis pigmentosa 1-like 1 protein (RP1L1), phosphodiesterase 6A (PDE6A), phosphodiesterase 6G (PDE6G), interphotoreceptor matrix proteoglycan 2 (IMPG2), sodium/potassium/calcium exchanger 1 or NCKX1 (SLC24A1), and potassium voltage-gated channel subfamily B member 1 (KCNB1). Other proteins involving synapse function were also differentially phosphorylated, such as protein unc-119 homolog A (UNC119), SNARE-associated protein Snapin (SNAPIN), synapsin-2 (SYN2), and disks large-associated protein 1 (DLGAP1). Furthermore, structural and cilia-related proteins were also differentially phosphorylated, including microtubule-actin cross-linking factor 1 (MACF1), microtubule-associated protein 1B (MAP1B), rootletin (CROCC), kinesin-like protein (KIF3A), spermatogenesis-associated protein 7 homolog (SPATA7), and jouberin (AHI1). Posttranslational modifications of these structural or cilia-related proteins may lead to the observed outer segment changes seen in Figure 2, C and D. In addition, phosphoglycerate mutase 1 (PGAM1) and glyceraldehyde-3-phosphate dehydrogenase (GAPDH), which are both essential enzymes involved in glycolysis, were differentially phosphorylated in *Prkaa2^-Rhod/-Rhod^* rod photoreceptors, suggesting a role in the upregulated glycolysis seen in Figure 3. Although AMPK has been described to regulate glycolysis through regulation of 6-phosphofructo-2-kinase (PFKFB) (7), we did not observe any significant changes in phosphorylation status of either PFKFB2 or PFKFB3. We plotted a selection of significantly downregulated phosphoproteins on a heatmap of *z* scores to visualize the spread of each sample (Figure 4D).

Closer examination of the phosphoproteomic data identified IMPDH as a strong candidate for the phenotype seen in PRKAA2-deficient mice, as IMPDH is the rate-limiting enzyme for de novo GMP synthesis. A previous study reported marked changes in retinal ATP and GTP as a result of IMPDH dysfunction (38). The authors also demonstrated that phosphorylation of the S416 moiety downregulated IMPDH activity. Our phosphoproteomics data showed that both IMPDH1 and IMPDH2 had downregulated phosphorylation at the S416 moiety only in *Prkaa2^-Rhod/-Rhod^*, suggesting aberrant activation of IMPDH as a possible mediator of rod photoreceptor dysfunction in the setting of *Prkaa2* deficiency (Figure 4, E and F).

### IMPDH activity is aberrantly upregulated in dark-adapted Prkaa2^-Rhod/-Rhod^ retinas.

**Figure 5:**
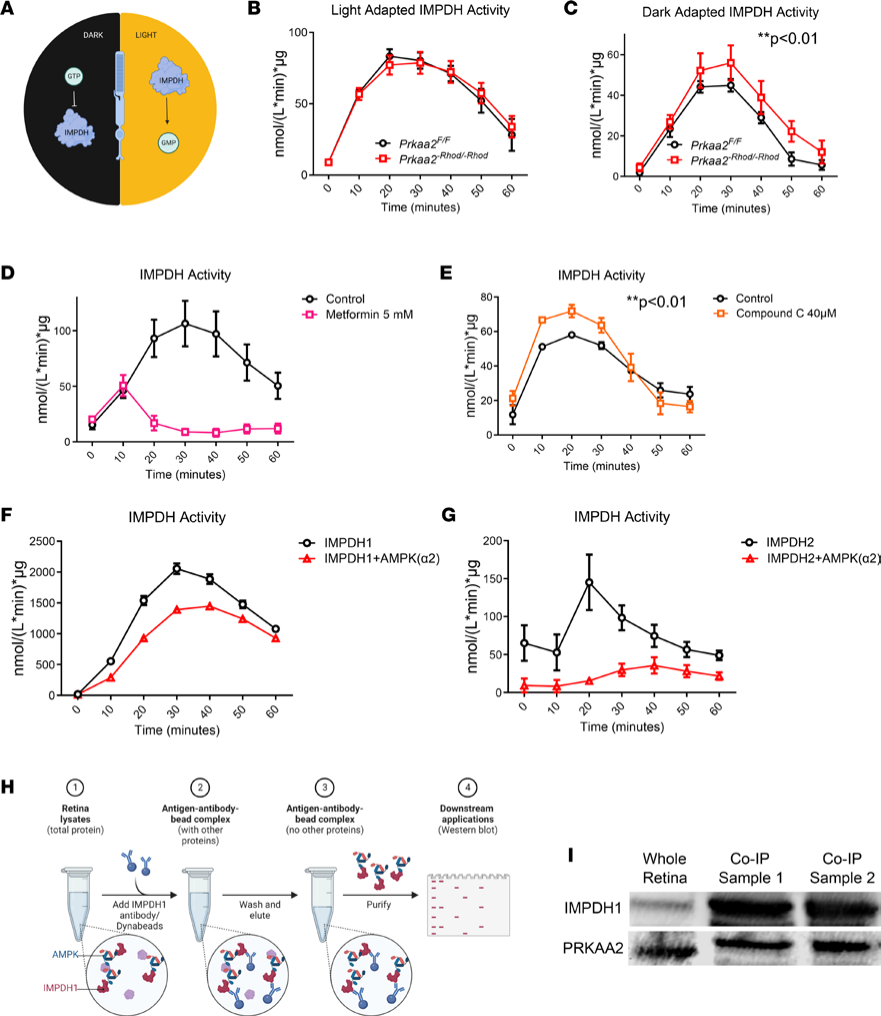
Dysfunctional PRKAA2 cannot regulate IMPDH, leading to IMPDH hyperactivity. (**A**) Schematic representation of IMPDH function in rod photoreceptors according to light exposure. Previous work has shown GTP allosterically inhibits IMPDH in dark-adapted conditions while IMPDH is active to produce GMP in light-adapted conditions. (**B**) Ambient light–adapted retinas from *Prkaa2^-Rhod/-Rhod^* were assessed for IMPDH activity (*n* = 4). No significant changes were detected. (**C**) Dark-adapted retinas from *Prkaa2^-Rhod/-Rhod^* showed significantly increased IMPDH activity (*n* = 6; **P < 0.01 by 2-way ANOVA). (**D**) Ambient light–adapted wild-type retinas were assessed for IMPDH activity after treatment with 5 mM metformin, an AMPK activator. Metformin is a slower activator of AMPK, and metformin treatment inhibits IMPDH activity compared with that of control retinas (*n* = 6, *P* < 0.0001 by 2-way ANOVA). (**E**) Dark-adapted wild-type retinas were assessed for IMPDH activity after treatment with 40 μM compound C, an AMPK inhibitor. Lysates treated with compound C exhibited significantly higher IMPDH activity compared with controls (*n* = 4, **P < 0.01 by 2-way ANOVA). (**F** and **G**) Recombinant human AMPK (α2β1γ1) was incubated with recombinant human (**F**) IMPDH1 or (**G**) IMPDH2 to assess the effect of AMPK on IMPDH activity. Both IMPDH1 and IMPDH2 when incubated with AMPK had significantly attenuated IMPDH activity compared with IMPDH incubated alone (*n* = 4, *P* < 0.0001 by 2-way ANOVA). (**H**) Schematic workflow of the co-immunoprecipitation protocol of IMPDH1. Six dark-adapted retinas from wild-type mice were dissected and lysed in extraction buffer. Dynabeads coated with IMPDH1 antibody were added to the suspension and allowed to bind to IMPDH1. Attached IMPDH1 along with bound proteins were isolated. The resulting eluant was then used for Western blots to detect IMPDH1 and bound protein. (**I**) The results of Western blot detection of co-immunoprecipitation samples are depicted. Clear bands representing IMPDH1 along with PRKAA2 demonstrate PRKAA2 was bound to IMPDH1 in wild-type retinas. Values are mean ± SEM.

Based on previous reports, IMPDH is regulated differently according to light conditions in rod photoreceptors (Figure 5A) (38). IMPDH is allosterically inhibited by increased GTP levels in dark-adapted conditions, while IMPDH is activated to produce GMP in light adapted conditions. To ascertain whether IMPDH is aberrantly activated in the *Prkaa2^-Rhod/-Rhod^* rod photoreceptors, we utilized a colorimetric plate-based assay to specifically measure IMPDH activity using dissected and lysed retinas from *Prkaa2^-Rhod/-Rhod^* mice. This assay is based on the reduction of the tetrazolium salt INT in an NADH-coupled reaction to INT-formazan, which exhibits a new absorption maximum at 492 nm and allows for sensitive measurement of IMPDH activity (39). When measuring IMPDH activity in ambient-light conditions, we detected no significant changes in *Prkaa2^-Rhod/-Rhod^* (Figure 5B). However, we hypothesized that IMPDH activity was constitutively activated in light conditions even in wild-type controls based on previous reports of IMPDH activation in rod photoreceptors (38). Therefore, we sought to investigate IMPDH activity in dark-adapted conditions. After overnight dark adaptation, *Prkaa2^-Rhod/-Rhod^* retinas were processed and measured for IMPDH activity. Indeed, the overall absolute values of IMPDH activity were considerably lower than in the light-adapted counterparts, and we were able to observe significantly higher levels of IMPDH activity in *Prkaa2^-Rhod/-Rhod^* retinas (Figure 5C). These data suggest that PRKAA2 interacts with and phosphorylates IMPDH1 and IMPDH2 to downregulate their activity, and PRKAA2 dysfunction leads to significant upregulation of IMPDH activity.

### AMPK-affecting compounds alter IMPDH activity.

We next investigated whether metformin, an AMPK activator, suppresses IMPDH activity in retinal lysates (Figure 5D). We measured IMPDH activity from retinal lysates of wild-type mice treated with metformin and observed a substantial abrogation of IMPDH activity in metformin-treated lysates. He et al. also demonstrated that the effect of AMPK activation by metformin in the retina is notably slower than other that of other AMPK activators (40), which corroborates the delay in IMPDH activity inhibition seen in our assay. These results suggest that a potent effect of metformin in the retina is inhibition of IMPDH activity, which could be through activation of AMPK.

We also sought to investigate whether compound C, or dorsomorphin, an AMPK inhibitor, upregulates IMPDH activity in retinal lysate (Figure 5E). Following our hypothesis on IMPDH activity based on dark adaptation, we assumed IMPDH activity is naturally inhibited by AMPK in dark-adapted retinas, so we sought to test the effect of AMPK inhibition in dark-adapted wild-type retinas. Indeed, we detected significantly upregulated IMPDH activity in lysates treated with compound C. Previous studies have noted 40 μM of compound C is sufficient for complete inhibition of AMPK activity; however, it is also noted that compound C can affect numerous other kinases (41). Therefore, we cannot rule out that other kinase inhibition may also affect IMPDH activity in addition to AMPK inhibition.

### IMPDH is a direct target of PRKAA2 in the retina.

To validate the effect of IMPDH regulation by PRKAA2, we used recombinant human AMPK (α2β1γ1) incubated with recombinant human IMPDH1 or IMPDH2 and measured IMPDH activity. We determined that β1 and γ1 were the highest expressed isoforms of the other AMPK subunits based on bulk RNA-sequencing data of the retina (19) and hypothesized that AMPK (α2β1γ1) would reduce IMPDH activity compared with IMPDH alone. We observed significantly reduced IMPDH activity in IMPDH incubated with AMPK (α2β1γ1) compared with IMPDH incubated alone (Figure 5, F and G).

We hypothesized that we could visualize PRKAA2 and IMPDH to be in the same subcellular compartment if they interact with each other. To test this, we used immunofluorescence to visualize localization of PRKAA2 and IMPDH in *Prkaa2^-Rhod/-Rhod^* retinal sections (Supplemental Figure 5). The knockout domain of PRKAA2 in *Prkaa2^-Rhod/-Rhod^* is contained within the kinase domain, which allowed us to utilize a PRKAA2 antibody that specifically targets an immunogen outside the kinase domain. Using this targeted approach, we identified that both isoforms of IMPDH were modestly appreciated in the same subcellular compartment as PRKAA2, especially in *Prkaa2^-Rhod/-Rhod^* sections, compared with *Prkaa2^fl/fl^* sections, particularly in the inner segment layers. These data suggest PRKAA2 may colocalize with IMPDH when the kinase activity is dysfunctional. One potential reason for this may be that PRKAA2 functionally “dead kinase” is actively attempting to inhibit hyperactivated IMPDH in the *Prkaa2^-Rhod/-Rhod^* rod photoreceptors.

We further sought to distinguish whether IMPDH dysregulation was either a direct or indirect manifestation of PRKAA2 abrogation. To elucidate whether IMPDH is a direct target of PRKAA2 in the retina, we performed co-immunoprecipitation experiments (Figure 5H). By co-immunoprecipitating IMPDH1, the predominantly expressed IMPDH isoform in the retina (19, 38), we investigated whether we could detect PRKAA2 bound to IMPDH1 through Western blotting. We utilized dark-adapted retinas from wild-type mice based on our hypothesis that PRKAA2 interacts with IMPDH more so in dark-adapted photoreceptors. Indeed, we were able to detect PRKAA2 in the IMPDH1 co-immunoprecipitated suspension, suggesting PRKAA2 directly binds to IMPDH1 in the retina (Figure 5I).

### Mycophenolate mofetil treatment improves Prkaa2^-Rhod/-Rhod^ visual function.

**Figure 6:**
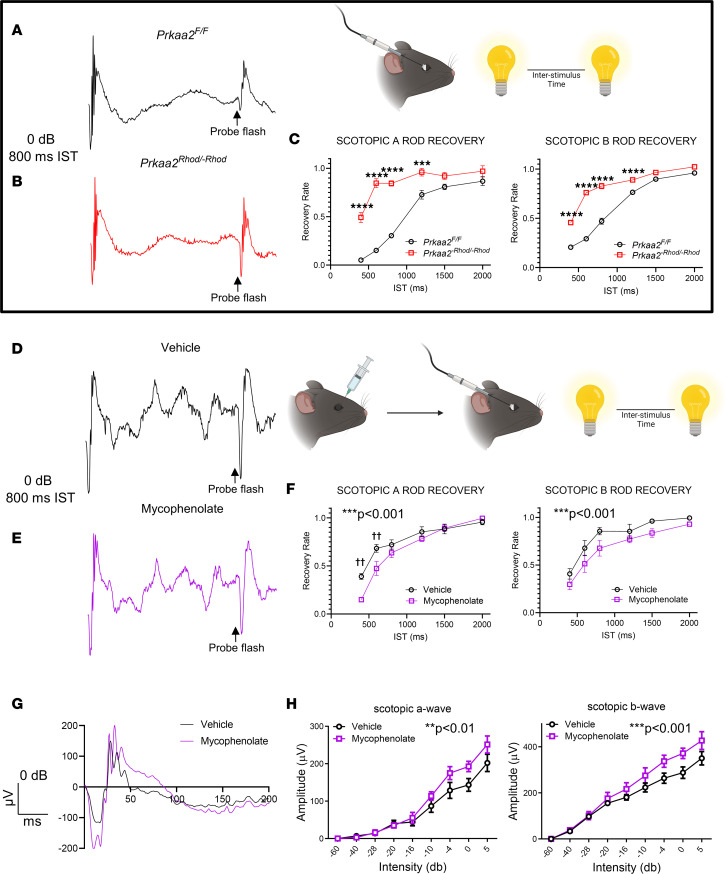
Mycophenolate mofetil treatment improves visual function deficits measured by electroretinography in *Prkaa2^-Rhod/-Rhod^*. (**A** and **B**) Representative electroretinography traces of *Prkaa2^-Rhod/-Rhod^* mass rod recovery. A 0 dB flash with 800 ms interstimulus time (IST) is represented. (**A**) A trace from *Prkaa2*^fl/fl^ shows typical attenuated scotopic a and b waves in the test response indicated by the probe flash with 800 ms interstimulus time. (**B**) A trace from *Prkaa2^-Rhod/-Rhod^* shows abnormally large scotopic a and b waves with 800 ms IST in the test response indicated by the probe flash. (**C**) Quantification of scotopic A and scotopic B rod recovery (*n* = 7). *Prkaa2^-Rhod/-Rhod^* mice show significantly faster rod recovery compared to wild-type littermates (***P < 0.001, ****P < 0.0001 by post hoc Bonferroni’s multiple comparisons test; *P* < 0.0001 by 2-way ANOVA for both graphs). (**D** and **E**) Representative electroretinography traces of *Prkaa2^-Rhod/-Rhod^* mass rod recovery from eyes treated with vehicle or mycophenolate mofetil. (**D**) Vehicle-treated eyes demonstrate similar waveform shapes as (**E**) mycophenolate-treated eyes from the probe flash. (**F**) Quantification of scotopic a and scotopic b rod recovery (*n* = 5). Mycophenolate-treated eyes show significantly slower scotopic a and scotopic b rod recovery compared with vehicle-treated eyes (^††^*P* < 0.01 by post hoc Bonferroni’s multiple comparisons test; ***P < 0.001 by 2-way ANOVA). (**G**) Representative traces of full-field scotopic electroretinography from vehicle- and mycophenolate-treated eyes. Although the waveform shape is slightly altered, mycophenolate treatment improves scotopic a and scotopic b wave amplitudes compared with vehicle treatment. (**H**) Quantification of scotopic a and scotopic b wave amplitudes from full-field scotopic electroretinography (*n* = 5). Mycophenolate-treated eyes had significantly improved scotopic a and scotopic b wave amplitudes compared with vehicle-treated eyes (**P < 0.01, ***P < 0.001 by 2-way ANOVA). Values are mean ± SEM.

Previous studies have examined the in vivo effect of IMPDH dysregulation using the dual-flash paradigm of electroretinography (38, 42). As our previous data have demonstrated IMPDH is dysregulated in *Prkaa2^-Rhod/-Rhod^*, we hypothesized that *Prkaa2^-Rhod/-Rhod^* exhibit altered mass rod recovery as measured by the dual-flash paradigm due to changed IMPDH activity and potentially changed GTP/cGMP levels. Indeed, using this paradigm, *Prkaa2^-Rhod/-Rhod^* demonstrated significantly faster rod recovery (Figure 6, A–C). Following these experiments, we sought to test if these changes were reversible. We utilized mycophenolate mofetil, a known IMPDH inhibitor, injected intravitreally into the eyes of *Prkaa2^-Rhod/-Rhod^* and assessed the mass rod recovery and full-field scotopic electroretinography function (38). Left eyes were injected with PBS vehicle while right eyes were injected with 1 μL of 10 mM mycophenolate mofetil to produce an estimated effective concentration of 2 mM in the eye. Mass rod recovery was slower in the mycophenolate-injected eyes compared with vehicle-injected eyes (Figure 6, D–F). However, the full-field scotopic electroretinography measurements yielded significantly improved scotopic a wave and scotopic b wave amplitudes (Figure 6, G and H). Furthermore, injection of the same concentration of mycophenolate in wild-type mice did not improve visual function, suggesting the effects of mycophenolate-induced visual function improvement is specific to *Prkaa2^-Rhod/-Rhod^* (Supplemental Figure 6). These data suggest IMPDH hyperactivity in *Prkaa2^-Rhod/-Rhod^* is suppressible, and reducing IMPDH hyperactivity improves the function of rod photoreceptors.

## Discussion

Our study suggests that AMPK, specifically through PRKAA2, regulates IMPDH activity in rod photoreceptors. First, we showed that the catalytic subunits of AMPK, PRKAA1 and PRKAA2, are both expressed in the neurosensory retina. In addition, these subunits exhibit distinct roles in that only PRKAA2 plays a unique functional role in rod photoreceptor neuron function during homeostasis as measured by electroretinography. We provide evidence that PRKAA2 directly binds and acts on IMPDH and that through this interaction, AMPK may regulate the activity of IMPDH, the rate-limiting enzyme for GMP synthesis. Although the phosphorylation site of S416 of IMPDH has been described as a consensus site for AMPK (43), our study is the first to our knowledge to demonstrate that AMPK regulates IMPDH in vivo specifically in retinal neurons. Disruption of the AMPK/IMPDH axis with IMPDH hyperactivity contributes to visual function deficits as mycophenolate-induced IMPDH inhibition ameliorates visual function deficits seen in *Prkaa2^-Rhod/-Rhod^* mice.

IMPDH inhibition by AMPK suggests AMPK promotes the de novo synthesis of AMP by inhibiting de novo synthesis of GMP, which would allow more AMP as substrate to produce ATP through glycolysis and oxidative phosphorylation. However, there are likely many intermediate factors that contribute to maintenance of steady-state AMP and GMP levels as *Prkaa2^-Rhod/-Rhod^* retinas did not exhibit changes to AMP and GMP levels despite evidence of increased IMPDH activity. Further investigation is warranted to link increased de novo production of AMP to increased levels of ATP as AMPK is canonically associated with increasing ATP levels. Additional studies are also necessary to establish the link between the presumed increased production in GMP and the observed increase in cGMP and GTP levels in *Prkaa2^-Rhod/-Rhod^* retinas.

The mass rod recovery electroretinography data from *Prkaa2^-Rhod/-Rhod^* may hint at changes to the phototransduction cascade. Lyubarsky and Pugh (42) hypothesized that changes in mass rod recovery through the dual-flash paradigm could be attributed to either rare long-lived rhodopsin inactivation or the availability of GTP-bound transducin-α to bind PDE_γ_. In tandem with Lyubarsky and Pugh’s hypothesis, the findings from Plana-Bonamaisó et al. that demonstrate inhibiting de novo GMP synthesis leads to slower mass rod recovery may suggest mass rod recovery is influenced by levels of guanine-phosphate levels. This may potentially be due to availability of GTP-bound transducin-α or cGMP levels. Considering the metabolomics data suggesting increased GTP levels in *Prkaa2^-Rhod/-Rhod^*, we speculate that increased GTP or cGMP levels lead to faster mass rod recovery, which reflects the opposite phenotype seen in the inhibition of IMPDH (38). Increased levels of cGMP may also contribute to the visual function defects seen in *Prkaa2^-Rhod/-Rhod^*. Major players in the phototransduction cascade, such as phosphodiesterase and guanylyl cyclase, consume and produce cGMP, respectively, to delicately regulate cGMP levels and cyclic nucleotide gated channels (44). Additional studies are needed to elucidate the mechanism of the potential effect of cGMP levels on visual function in *Prkaa2^-Rhod/-Rhod^* as there likely are many factors contributing to this effect; however, candidate proteins that may have aberrant activity related to cGMP regulation and closely associated calcium regulation include phosphodiesterase 6A (PDE6A), phosphodiesterase 6G (PDE6G), and sodium/potassium/calcium exchanger 1 or NCKX1 (SLC24A1), as they all have altered phosphorylation based on phosphoproteomics data. Perturbed activity of these enzymes may contribute to disturbed cGMP/GTP homeostasis as seen in *Prkaa2^-Rhod/-Rhod^* retinas as PDE6A and PDE6G directly converts cGMP to GMP and NCKX1 contributes to intracellular calcium levels, which affects guanylyl cyclase activity converting GTP to cGMP.

Although AMPK putatively upregulates catabolic processes (1), we observed an increase in glycolysis along with increased ATP levels with loss of PRKAA2 activity. We can therefore speculate that PRKAA2 function is not necessary to upregulate glycolysis. Furthermore, glycolytic upregulation in *Prkaa2^-Rhod/-Rhod^* may be controlled by PRKAA1 or an AMPK-independent pathway. The mechanism of increased glycolysis in *Prkaa2^-Rhod/-Rhod^* has yet to be elucidated. However, our phosphoproteomics data yielded interesting insights into which glycolysis-related proteins may be affecting the glycolysis phenotype in *Prkaa2^-Rhod/-Rhod^*. Both PGAM1 and GAPDH had markedly different phosphorylation statuses, suggesting either by direct or indirect effect of *Prkaa2* knockout that PGAM1 and GAPDH may be affecting the glycolysis phenotype. Surprisingly, we did not observe differential phosphorylation status of either PFKFB2 or PFKFB3 despite their being described targets of AMPK for glycolytic regulation (7). These results highlight how, despite the thorough characterization of AMPK function in previous studies, the putative targets of AMPK are perhaps regulated according to distinct isoform function and tissue context.

By investigating the *Prkaa1^-Rhod/-Rhod^* and *Prkaa2^-Rhod/-Rhod^* models, we have characterized a novel role for the PRKAA2 catalytic isoform of AMPK in regulating IMPDH activity. Mutations in *IMPDH1* in humans are associated with severe forms of inherited blindness, with 9 known mutations linked to autosomal dominant retinitis pigmentosa-10 (RP10) (45–49) and 2 known mutations linked to Lebers congenital amaurosis type 11 (46). Interestingly, RP10 mutations have been suggested to be gain-of-function mutations, as *Impdh1*-knockout mice present mild retinopathy (50). Given that *Prkaa2^-Rhod/-Rhod^* retinas recapitulate features of *Impdh1* gain-of-function mutations, these mice could present a suitable model to study and treat RP10-like mutations.

The results of our study warrant further investigation into whether PRKAA2 regulates IMPDH activity in other cell types, particularly ones with high energetic demands and those that predominantly express *Prkaa2* over *Prkaa1*. However, it is also possible that the effect of IMPDH activity is more strongly appreciated in rod photoreceptors because they uniquely rely on cGMP and the other guanosine phosphates for phototransduction and dark current maintenance. This study was largely limited to observing the effects of PRKAA2 in homeostasis; therefore, further investigation is necessary to determine the functions of both PRKAA1 and PRKAA2 in disease. Previous studies have described a protective role of AMPK based on both loss-of-function and gain-of-function studies in both endotoxin-induced uveitis and light-induced retinal degeneration models (3, 16), but the role of the individual catalytic subunits in disease is unclear. Future studies that examine specific pathways regulated by either PRKAA1 or PRKAA2 in photoreceptors during disease will illuminate our understanding of how AMPK regulates cellular energy balance and function in disease models.

## Methods

### Animals.

All animal experiments were conducted in accordance with the Association for Research in Vision and Ophthalmology Statement for the Use of Animals in Ophthalmic and Vision Research and Washington University School of Medicine in St. Louis Animal Care and Use guidelines and after approval by the Institutional Animal Care and Use Committee (IACUC). Male mice between 8 and 12 weeks old (2–3 months of age) were used in this study. Mice were housed in a 12-hour light/12-hour dark cycle with free access to food and water. *Prkaa1^fl/fl^* and *Prkaa2^fl/fl^* mice were previously characterized (34) and purchased from The Jackson Laboratory (014141 and 014142). These mice were crossed with mice carrying 1 copy of the rhodopsin-iCre75 transgene, which were provided by Ching-Kang Jason Chen, UT Health San Antonio, San Antonio, Texas, USA, and have been previously characterized (51), to generate *Prkaa1^-Rhod/-Rhod^* and *Prkaa2^-Rhod/-Rhod^*. We confirmed that these mice did not carry the *Crb1* gene *rd8* mutation (data not shown). Mice were fully backcrossed to the inbred C57BL/6J background.

### Single-cell RNA-sequencing analysis.

Published data sets were imported into Monocle 3 (v1.3.1) (52) for reanalysis and confirmation of published cell type calls. Count matrix was then exported using the exprs() function before normalization of counts to 10K (TP10K) and computation of average expression of genes of interest within each cell type. Nominal 0.01 TP10K pseudocount was added to each average count per cell type before calculation of ratio of PRKAA1 to PRKAA2.

### Immunomagnetic rod photoreceptor isolation.

To isolate rod photoreceptors from the retina, we utilized the Papain Dissociation System (Worthington Biochemical Corporation) following a previously described modified protocol (26), followed by EasySep Mouse PE and Biotin positive selection kit (Stem Cell Technologies) following the manufacturer’s protocol. We used 2 μg/mL of PE-conjugated anti-CD73 antibody (eBioscience catalog 12-0731-82) and 2 μg/mL of biotin-conjugated anti-rhodopsin antibody (Novus Biologicals catalog NBP1-47602B) as part of the EasySep protocol.

### RNA isolation and qRT-PCR.

Total RNA was extracted using RNeasy Micro Plus Kit (QIAGEN) according to the manufacturer’s instructions. To synthesize cDNA, total RNA was added to the High Capacity cDNA Reverse Transcription Kit (Thermo Fisher Scientific) and reverse-transcribed according to the manufacturer’s instructions. qRT-PCR was performed in duplicate using the StepOnePlus Real Time PCR system (Thermo Fisher Scientific) using TaqMan Real-Time PCR Assays (Thermo Fisher Scientific), and the mRNA was quantified using the ΔΔCT method with *Gapdh* or *Actb* as the internal control. Custom TaqMan probes (Thermo Fisher Scientific) were created and used to specifically measure expression of exon 3 of *Prkaa1* and exon 2 of *Prkaa2* to confirm knockout in the respective models.

### Transmission electron microscopy.

We performed transmission electron microscopy as previously described (35). For ultrastructural analyses, eyecups were fixed in 2% paraformaldehyde/2.5% glutaraldehyde (Polysciences Inc.) in 100 mM sodium cacodylate buffer, pH 7.2, for 2 hours at room temperature and then overnight at 4°C. Samples were washed in sodium cacodylate buffer at room temperature and postfixed in 1% osmium tetroxide (Polysciences Inc.) for 1 hour. Samples were then rinsed extensively in double-distilled H_2_O prior to en bloc staining with 1% aqueous uranyl acetate (Ted Pella Inc.) for 1 hour. Following several rinses in double-distilled H_2_O, samples were dehydrated in a graded series of ethanol and embedded in Eponate 12 resin (Ted Pella Inc.). Sections of 95 nm were cut with an Ultracut UCT ultramicrotome (Leica Microsystems Inc.), stained with uranyl acetate and lead citrate, and viewed on a 1200 EX transmission electron microscope (JEOL USA Inc.) equipped with an 8-megapixel digital camera and Image Capture Engine V602 software (both Advanced Microscopy Techniques).

### Electroretinography.

Full-field scotopic electroretinography was performed as previously described (35). A UTAS BigShot System (LKC Technologies Inc.) was used. Mice were dark-adapted overnight. Under red light illumination, mice were anesthetized with an intraperitoneal injection of 86.9 mg/kg ketamine and 13.4 mg/kg xylazine. Pupils were dilated with 1% tropicamide eye drops. Body temperature was maintained at 37°C with a heating pad. Contact lens electrodes were placed bilaterally with appropriate reference and ground electrodes. The stimulus consisted of a full-field white light flash (10 μs). Raw data were processed using MATLAB software (MathWorks). The amplitude of the a wave was measured from the average pretrial baseline to the most negative point of the average trace, and the b wave amplitude was measured from that point to the highest positive point.

The mass rod recovery or dual-flash paradigm was utilized as previously described (38, 42). Briefly, mice underwent the same pipeline for full-field scotopic electroretinography. However, mice were instead exposed to sequential stimuli of a full-field white-light flash (10 μs, 0.977 cd·s/m^2^) of different ISTs per step. The a wave and b wave amplitudes of both the baseline flash and the probe flash were measured for each IST, and the ratio of the response to the probe flash to baseline flash was calculated.

### Intravitreal injection.

Mice were dark-adapted overnight. Under dim-red lighting, mice were anesthetized with an intraperitoneal injection of 86.9 mg/kg ketamine and 13.4 mg/kg xylazine. Pupils were dilated with 1% tropicamide eye drops. A 33-gauge Hamilton syringe was used to inject into the vitreous cavity. Left eyes were injected with 1 μL of PBS vehicle while right eyes were injected with 1 μL of 10 mM mycophenolate mofetil to produce an effective concentration of 2 mM in the eye. These mice then immediately underwent the electroretinography pipeline.

### Metabolomics measurement.

Mice were euthanized and retinas were immediately extracted and frozen in liquid N_2_. On the day of extraction, retinal tissues were homogenized in 160 μL of cold 50% MeOH solution in water using homogenizer (Branson) and then centrifuged (15,000*g*, 4°C, 10 minutes). Clear supernatant was transferred to a new tube containing 100 μL chloroform and vigorously shaken, then centrifuged (15,000*g*, 4°C, 10 minutes). The chloroform extraction was repeated 3 times. The clear aqueous phase (120 μL) was transferred to new tube and then lyophilized and stored at −80°C until measurement. Lyophilized samples were reconstituted with 60 μL of 5 mM ammonium formate (MilliporeSigma) and centrifuged at 12,000*g* for 10 minutes. Cleared supernatant was transferred to a sample tray. Serial dilutions of standards for each metabolite in 5 mM ammonium formate were used to determine the retention time and concentration. Liquid chromatography was performed by HPLC (1290; Agilent) with Atlantis T3 (LC 2.1 × 150 mm, 3 μm; Waters) (38). For steady-state metabolite analysis, 20 μL of samples were injected at a flow rate of 0.7 mL/min with 50 mM ammonium acetate and 5 mM medronic acid for mobile phase A and 100% acetonitrile for mobile phase B. Metabolites were eluted with gradients of 0–0.5 minutes 80%, 0.5–7.5 minutes 80%–70%, 7.5–8.5 minutes 70%–50%, 8.5–9 minutes 50%, 9–9.5 minutes 50%–80%, 9.5–11 minutes 80% of B. The metabolites were detected with a triple-quadrupole mass spectrometer (6460, Agilent) under positive ESI multiple reaction monitoring using *m/z* for AMP 348→136, ATP 508→136, GMP 364→152, GDP 444→152, GTP 524→152, cGMP 346→152, IMP 349→137. Metabolites were quantified by MassHunter quantitative analysis tool (Agilent) with standard curves and normalized by the protein amount in the sample.

### Extracellular flux analyses.

Oxygen consumption rate (OCR) and extracellular acidification rate (ECAR) measurements were used to evaluate the activity of oxidative phosphorylation and glycolysis, respectively, in retinas. Performing extracellular flux analyses using 1 mm biopsy punches of retinas was previously described (36). Mice were euthanized and retinas were extracted into Seahorse XF DMEM and maintained on a heating plate set to 37°C. Three 1 mm biopsy punches (Integra) were isolated from each retina of the central region sparing the optic nerve head. Retina punches were transferred to Seahorse XF24 Islet Capture microplates. Retina punches were then gently placed on the bottom of wells, and mesh inserts were affixed on top of retina punches. Each well contained either a single retina punch or no punches to use as background.The Seahorse XF24 sensor cartridge was hydrated with Seahorse XF Calibrant solution (1 mL per well) and placed in a non-CO_2_ incubator at 37°C the night before. One hour prior to the experiment, standard medium was removed and replaced with 500 μL prewarmed Seahorse XF DMEM 789 medium supplemented with 1 mM sodium pyruvate, 2 mM glutamine, and 10 mM glucose, pH 7.4, and placed in a non-CO_2_ incubator at 37°C. Chemicals from either the Mito Stress Test kit for OCR or Glycolysis Stress Test kit for ECAR were loaded into the sensor cartridge as previously described. Following calibration of the sensor cartridge in the XFe24 Extracellular Flux Analyzer, the cell plate was inserted. Each cycle consisted of 3 minutes of mixing and a 2-minute pause, followed by a 3-minute measurement period. Each cycle repeated 3 times. Total protein content of each well was measured using Pierce BCA Protein Assay Kit (Thermo Fisher Scientific) and used to normalize readings.

### Retina lactate excretion measurement.

Mice were euthanized and retinas were extracted and plated in a 12-well plate with 500 μL of Ames’ Media (prepared from 10 mL double-distilled H_2_O with 88 mg lyophilized Ames’ media, 19 mg sodium bicarbonate, and 20 mg glucose). Retinas were then incubated in a humidified incubator at 37°C with 5% CO_2_ for 30 minutes. The supernatants were then collected and quantified for lactate using a colorimetric assay (MilliporeSigma) following the manufacturer’s protocol.

### Phosphoproteomics.

Phosphoproteomics of isolated rod photoreceptors was performed as described previously (53). Mass spectrometric data were collected on an Orbitrap Fusion Lumos mass spectrometer in line with a Proxeon NanoLC-1200 UHPLC. The 100 μm capillary column was packed with 35 cm of Accucore 150 resin (2.6 μm, 150 Å; Thermo Fisher Scientific). Spectra were converted to mzXML using a modified version of ReAdW.exe. Database searching included all entries from the Mouse Genome Database. This database was concatenated with one composed of all protein sequences in the reversed order. Searches were performed using a 50 ppm precursor ion tolerance for total protein-level profiling. The product ion tolerance was set to 0.9 Da. These wide mass tolerance windows were chosen to maximize sensitivity in conjunction with SEQUEST searches and linear discriminant analysis. TMT tags on lysine residues and peptide N termini (+229.163 Da) and the carbamidomethylation of cysteine residues (+57.021 Da) were set as static modifications, while the oxidation of methionine residues (+15.995 Da) was set as a variable modification. For phosphorylation analysis, deamidation (+0.984) on asparagine and glutamine and phosphorylation (+79.966) on serine, threonine, and tyrosine were set as variable modifications. Peptide-spectrum matches (PSMs) were adjusted to a 1% false discovery rate (FDR). PSM filtering was performed using a linear discriminant analysis, as described previously (54) and then assembled further to a final protein-level FDR of 1%. Phosphorylation site localization was determined using the AScore algorithm. AScore is a probability-based approach for high-throughput protein phosphorylation site localization. Specifically, a threshold of 13 corresponded to 95% confidence in site localization. Proteins were quantified by summing reporter ion counts across all matching PSMs, as described previously. Reporter ion intensities were adjusted to correct for the isotopic impurities of the different TMT reagents according to manufacturer specifications. The signal-to-noise measurements of peptides assigned to each protein were summed, and these values were normalized so that the sum of the signal for all proteins in each channel was equivalent to account for equal protein loading. Last, each protein was scaled such that the summed signal-to-noise for that protein across all channels was greater than 100, thereby generating a relative abundance measurement.

### Immunofluorescence histology.

Retinal sections fixed in 4% paraformaldehyde in 1× PBS were deparaffinized, then blocking and hyperpermeabilization were performed through PBS containing 5% bovine serum albumin and 0.1% Triton X-100 for 1 hour. Sections were then incubated in blocking buffer with anti-PRKAA2 (R&D Systems, catalog AF2850; 1:100) and either anti-IMPDH1 (Proteintech, catalog 220921AP; 1:100) or anti-IMPDH2 (Proteintech, catalog 12948-1-AP; 1:100) antibodies overnight at 4°C. The next day, sections were rinsed in PBS and subsequently incubated with Alexa Fluor dyes corresponding to the fluorescence-conjugated secondary antibodies at room temperature for 1 hour. Nuclei were counterstained with DAPI (MilliporeSigma). Images were taken with the Zeiss LSM 800 Confocal Laser Scanning Microscope.

### In situ hybridization.

Formalin-fixed and dehydrated slides were pretreated with RNAscope protease reagents (Advanced Cell Diagnostics). Either *Prkaa1* or *Prkaa2* probes were added on each slide and incubated at 40°C for 2 hours in a HybEZ Oven (Advanced Cell Diagnostics). *Prkaa1* probes were customized to target exon 3 while *Prkaa2* probes were customized to target exon 2. Amplification steps and color development using RNAscope 2.5 HD detection reagents (Advanced Cell Diagnostics) were performed in accordance with the manufacturer’s instructions. After counterstaining with hematoxylin, coverslips were placed on slides. Images were taken with the Leica DMi8.

### IMPDH activity assay.

Mice were euthanized and retinas were extracted after either with or without dark adaptation overnight. Each sample was pooled from four retinas. These retinas were then lysed in lysis buffer (Biomedical Research Service and Clinical Application) and the IMPDH activity assay (Biomedical Research Service and Clinical Application) was utilized in accordance with the manufacturer’s protocol. The manufacturer’s protocol for the IMPDH activity assay can be found here: https://www.bmrservice.com/impdhassay.html Briefly, lysed retina samples were applied in duplicates into a 96-well plate with 1 replicate serving as a control well and the other a reaction well. Both wells received IMPDH assay solution, but only the reaction wells received IMPDH substrate. The combined assay solution was added rapidly to each well containing retinal lysate samples. The plate was kept on ice during the addition of all reagents. The plate was then swiftly transported to a preheated microplate reader at 37°C where the assay began at time 0 when the plate was inserted and immediately scanned for colorimetric absorption of 492 nm. The microplate reader then measured the same absorption maximum every 10 minutes for 60 minutes. After compiling all the measurements, enzyme activity was calculated with the difference of absorption measurement between control and reaction wells at each time point. The equation to calculate enzyme activity is as follows: ΔOD × 1,000 × 70 μL/(incubation time in minutes × 0.5 cm × 18 × 20 μL) × μg = μmol/(L × minutes) × μg. With incubation time being the only changing independent variable at each time point of measurement, a new constant was used at each time point to calculate the enzyme activity. In this assay, activity is measured as a function of absorption difference between the control and reaction wells, so activity can decrease as the difference between the control and reaction wells become smaller. In other words, the absorption measurement increase is not specific to IMPDH activity, so IMPDH activity is calculated relatively with the addition of IMPDH substrate. The assay used in our study uses the principle of measuring a new absorption maximum made through the reduction of tetrazolium salt INT in an NADH-coupled enzymatic reaction to INT-formazan as IMPDH activity reduces NAD^+^ to NADH. The assay was carried out under dim-red light with dark-adapted samples protected from white light throughout the entire assay.

Assays comparing IMPDH activity from retinas of *Prkaa2^-Rhod/-Rhod^* were performed according to the manufacturer’s protocol as described above. Assays assessing the effect of metformin (MilliporeSigma) were performed using ambient light–adapted retinas from wild-type mice and added to produce an effective concentration of 5 mM in each well. Assays assessing the effect of Compound C (MilliporeSigma) were performed using overnight dark-adapted retinas from wild-type mice and added to produce an effective concentration of 40 μM in each well. Assays assessing the effect of recombinant AMPK (Promega) on recombinant IMPDH1 (R&D Systems) or IMPDH2 (R&D Systems) were performed as follows: 500 ng IMPDH1 or 500 ng IMPDH2, 500 μM dithiothreitol, 1× reaction buffer A (Promega), 100 μM AMP solution (Promega), and either PBS or 500 ng AMPK and PBS were combined to constitute the initial solution before addition of the IMPDH activity assay constituents.

### Co-immunoprecipitation.

Co-immunoprecipitation of IMPDH1 from wild-type retinas was performed using the Dynabeads Co-Immunoprecipitation Kit (Thermo Fisher Scientific) in accordance with the manufacturer’s protocol. We used 1.5 mg of Dynabeads to bind each sample from 6 total lysed retinas. We used 10 μg of IMPDH1 antibody (BioLegend) for each sample.

### Immunoblotting.

Resultant samples from co-immunoprecipitation were loaded into lanes for SDS-PAGE. We loaded 1 μg of samples into each lane, and 1 lane of 20 μg of lysed total retinas was used as control. The proteins were then transferred onto a nitrocellulose membrane (0.22 μm pore size). Membranes were blocked with 5% bovine serum albumin in PBS. Membranes were then incubated with primary antibodies overnight at 4°C, followed by secondary antibodies for 1 hour at room temperature. IMPDH1 (Proteintech catalog 22092-1-AP) and PRKAA2 (R&D Systems catalog AF2850) were used for primary antibody incubation. Bands were visualized using the dual-channel Odyssey CLx Imaging System, and we quantified protein bands of interest using Image Studio.

### Sex as a biological variable.

Only male mice were used for experiments as interventions not included in the manuscript such as high-fat diet and streptozotocin-induced diabetes are more feasible for survival in male mice. Although their data were not included in the manuscript, male mice were used for consistency. The findings in the manuscript are expected to be relevant to more than one sex.

### Statistics.

All experiments except for the metabolomics and phosphoproteomics experiments were performed more than 2 times for replicates. In experimental groups of mice, littermates of the same sex were randomly assigned. Sample size was estimated by Sample Size Calculator (ClinCalc). Statistical analysis was performed using GraphPad Prism. A 2-tailed, unpaired Welch’s *t* test was utilized for a comparison between 2 groups. To compare 3 or more means, a 2-way ANOVA with the Bonferroni post hoc test was used to compare 2 or more means, respectively. *P* < 0.05 was considered statistically significant. Data were presented as bar or line graphs (mean ± SEM). When indicated, dot plots show each value.

### Study approval.

All animal experiments were reviewed and approved by the IACUC of Washington University in St. Louis and performed in accordance with the Washington University School of Medicine Animal Care and Use guidelines.

### Data availability.

The data sets used in this study can be found in online repositories. The accession numbers are as follows: National Center for Biotechnology Gene Expression Omnibus GSE127942 (mouse retina bulk RNA), GSE176069 (mouse hepatocyte bulk RNA), GSE125708 (mouse retina single-cell RNA), GSE135133 (human retina single-cell RNA), GSE63473 (mouse brain single-cell RNA), and GSE222647 (human PBMC single-cell RNA).

The mass spectrometry proteomics data have been deposited to the ProteomeXchange Consortium via the PRIDE partner repository with the data set identifiers PXD045667 (*Prkaa1^-Rhod/-Rhod^*) and PXD045669 (*Prkaa2^-Rhod/-Rhod^*).

This paper does not report original code. Any additional information required to reanalyze the data reported in this paper is available from the corresponding author upon request.

## Author contributions

TJL and RSA designed and analyzed the experiments. TJL, YS, PAR, NB, JBL, HTW, and AS conducted the experiments. TJL and RSA designed the methodology and wrote the manuscript. TJL and RSA conceptualized the study.

## Supplementary Material

Supplemental data

Supporting data values

## Version 1 01/16/2024

In-Press Preview

## Version 2 02/22/2024

Electronic publication
