# Supplementary material for: Catalytic isoforms of AMP-activated protein kinase differentially regulate IMPDH activity and photoreceptor neuron function
Source: JCI Insight. 2024 Jan 16;9(4):e173707. doi: 10.1172/jci.insight.173707 (PMC11143937; doi:10.1172/jci.insight.173707)
Supplement: Supplemental data [file jciinsight-9-173707-s102.pdf]

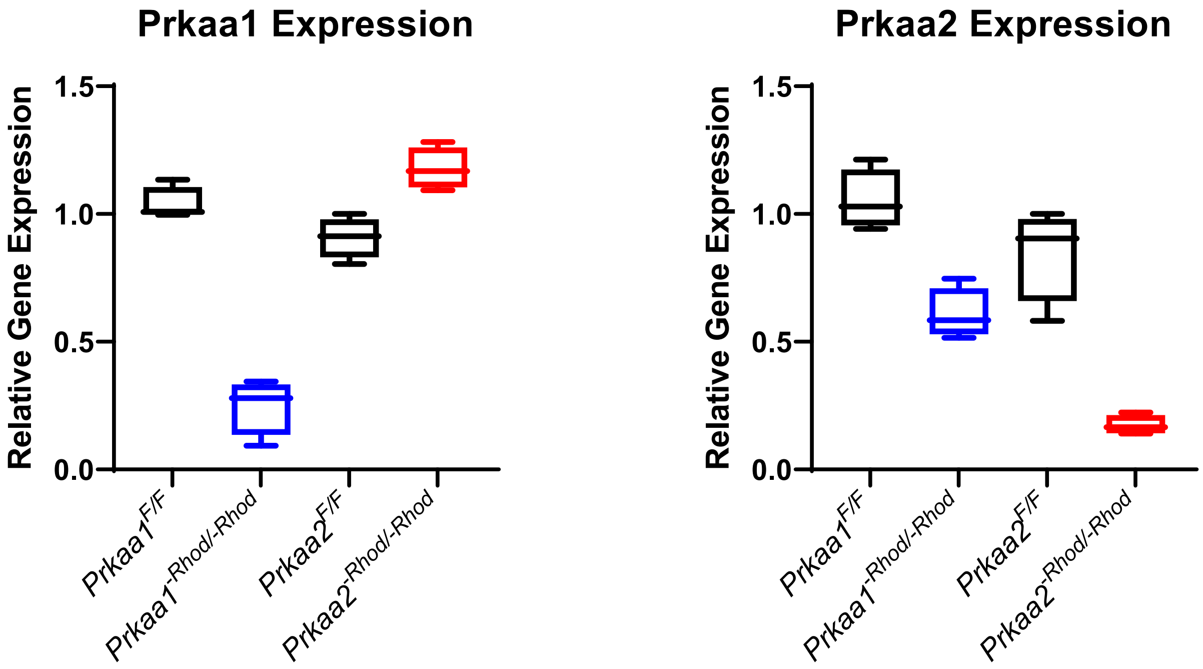

Supplementary Figure 1. *Prkaa1* and *Prkaa2* expression levels of isolated rod photoreceptors from *Prkaa1*$^{-Rhod/-Rhod}$ and *Prkaa2*$^{-Rhod/-Rhod}$. Knockout model expression levels were normalized to those of their corresponding wild-type cre negative littermates. *Prkaa1* expression was decreased in *Prkaa1*$^{-Rhod/-Rhod}$ compared to wild-type littermates while there were no drastic changes detected in *Prkaa2*$^{-Rhod/-Rhod}$ (n=4). *Prkaa2* expression was decreased in *Prkaa1*$^{-Rhod/-Rhod}$ with roughly 40% decreased expression while it was decreased in *Prkaa2*$^{-Rhod/-Rhod}$ by roughly 80% decreased expression (n=4). Although we see decreased expression of *Prkaa2* in *Prkaa1*$^{-Rhod/-Rhod}$, we did not observe structural or functional deficits in rod photoreceptors. Values are mean ± SE.

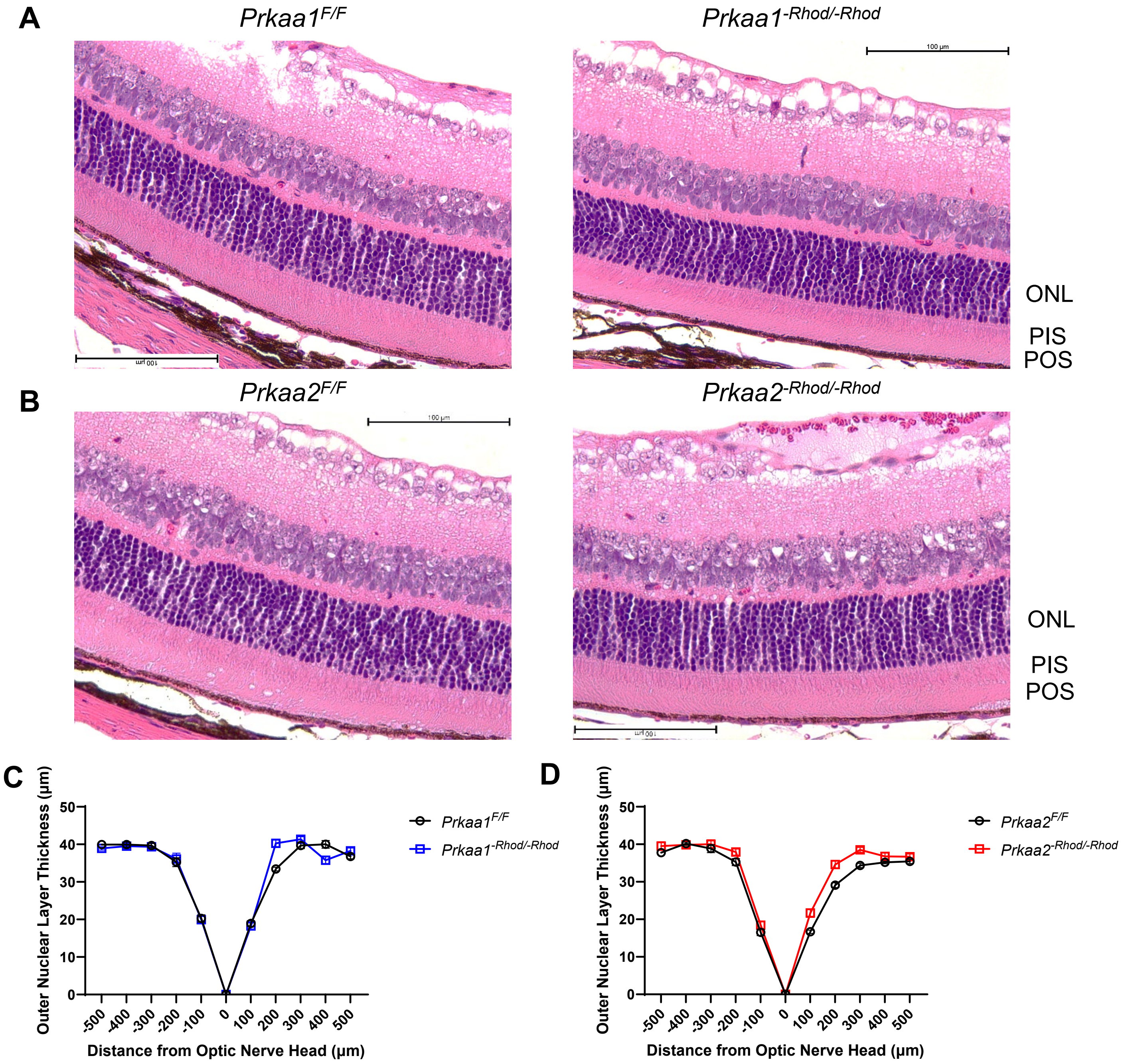

Supplementary Figure 2. Hematoxylin and eosin staining of *Prkaa1$^{-Rhod/-Rhod}$* and *Prkaa2$^{-Rhod/-Rhod}$*. No gross anatomical abnormalities were observed in neither **(A)** *Prkaa1$^{-Rhod/-Rhod}$* nor **(B)** *Prkaa2$^{-Rhod/-Rhod}$* rod photoreceptor layers such as the outer nuclear layer (ONL), photoreceptor inner segment (PIS), and photoreceptor outer segment layers (POS). No overt photoreceptor outer segment thinning was observed in *Prkaa2$^{-Rhod/-Rhod}$* despite electroretinography changes, suggesting rod photoreceptor dysfunction but not degeneration. **(C-D)** Butterfly plots quantifying the ONL thickness of both *Prkaa1$^{-Rhod/-Rhod}$* and *Prkaa2$^{-Rhod/-Rhod}$* compared to respective wild-type littermates confirm not significant differences in ONL thickness (n=12). Scale bars represent 100 µm. Values are mean ± SE.

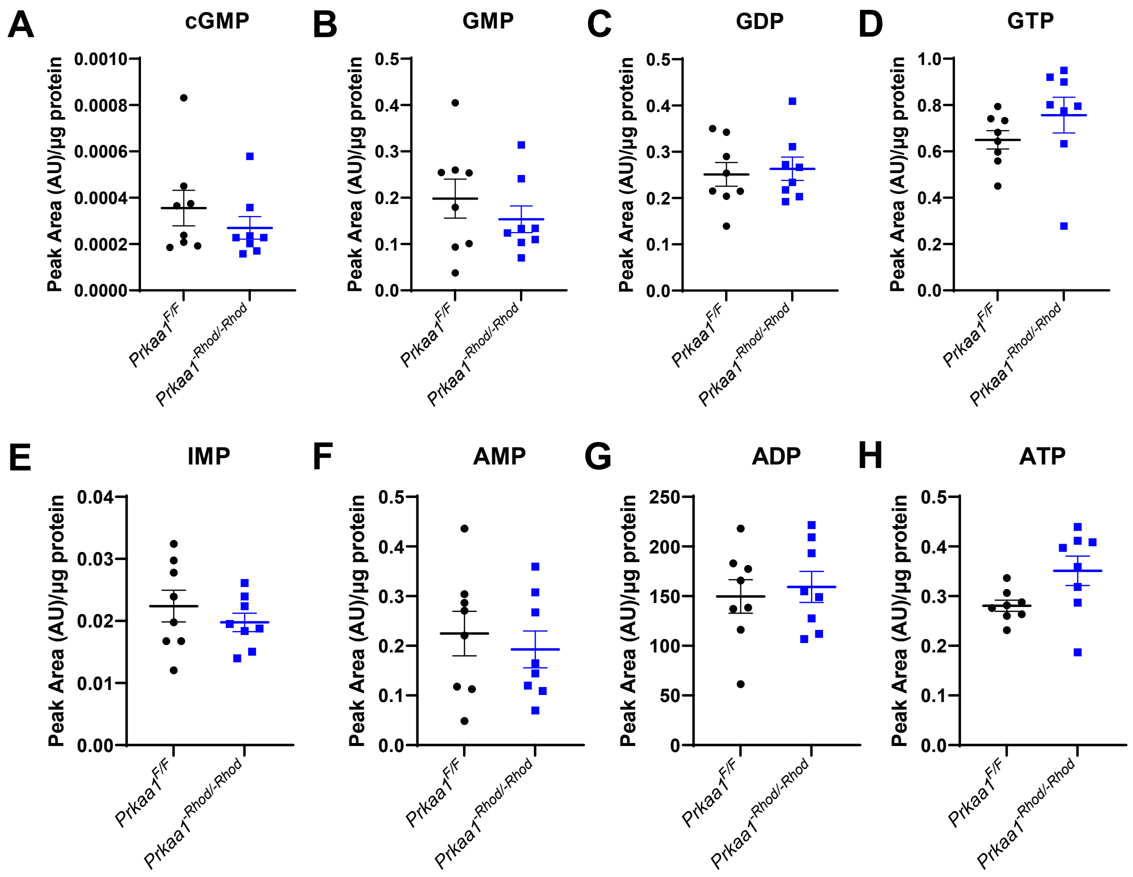

Supplementary Figure 3. Metabolomics of *Prkaa1$^{-Rhod/-Rhod}$* retinas. Retinas from *Prkaa1$^{-Rhod/-Rhod}$* and wild-type littermates underwent metabolomics measurement of phospho-purine quantities (n=8). No significant changes were observed in each measurement. Welch's t-test was used for each statistical measurement. Values are mean ± SE.

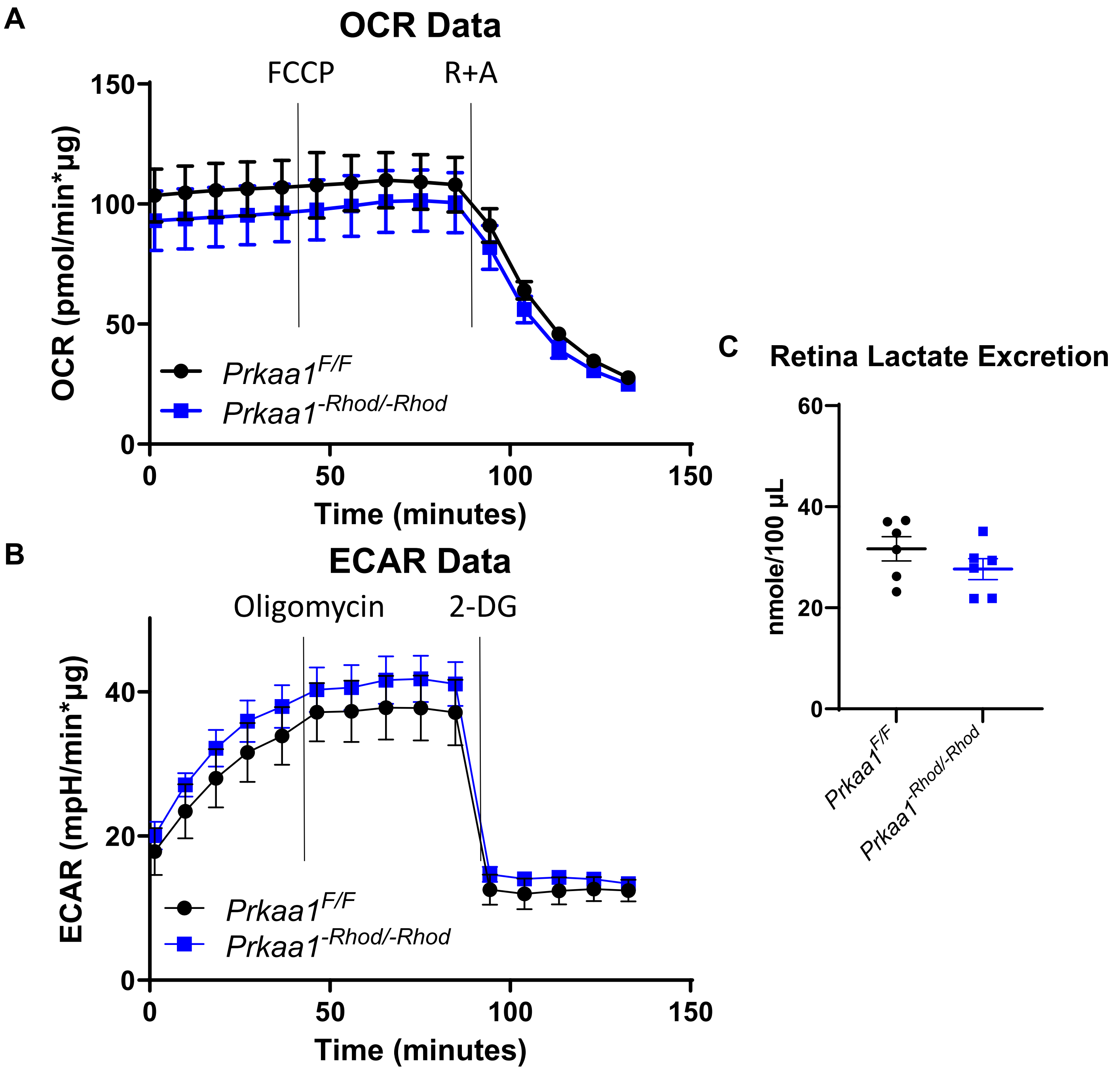

Supplementary Figure 4. Extracellular flux analyses of *Prkaa1*$^{-Rhod/-Rhod}$ retinas. (A-B) Oxidative phosphorylative flux and glycolytic flux were measured through oxygen consumption rate (OCR) (n=8) and extracellular acidification rate (ECAR) (n=7) respectively. No significant differences were detected in neither OCR nor ECAR suggesting *Prkaa1*$^{-Rhod/-Rhod}$ retinas did not exhibit changes in oxidative phosphorylative or glycolytic flux. Two-way ANOVA with post-hoc Bonferroni's multiple comparisons test were used for statistical analyses. **(C)** Lactate excretion measurements are a proxy measurement tool for photoreceptor glycolysis. Lactate measured from the supernatant of cultured retinas from *Prkaa1*$^{-Rhod/-Rhod}$ showed no significant differences compared to that of *Prkaa1*$^{F/F}$ (n=6). Values are mean ± SE.

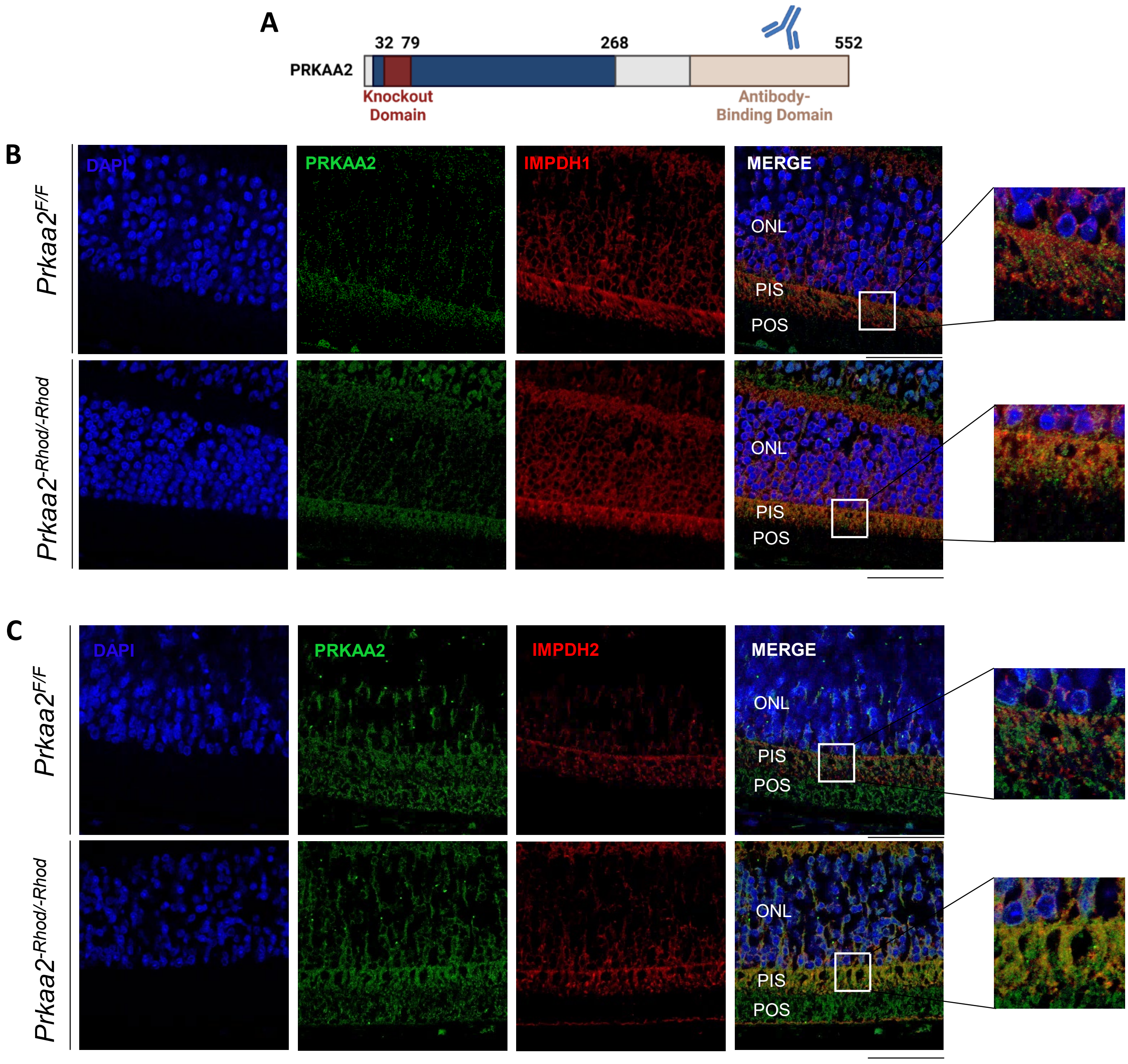

Supplementary Figure 5. PRKAA2 and IMPDH1/2 are appreciated in the same cellular compartment in *Prkaa2$^{-Rhod/-Rhod}$* inner segments compared to those of *Prkaa2$^{F/F}$*. (A) Schematic representation of the protein sequence of PRKAA2. The knockout domain of *Prkaa2$^{-Rhod/-Rhod}$* resides within aa 32 – aa 79. Antibodies with an immunogen targeting a separate region than the kinase domain can reliably bind to dysfunctional PRKAA2, such as the one we utilized. **(B-C)** Immunofluorescent staining of PRKAA2 and IMPDH1/2 of *Prkaa2$^{-Rhod/-Rhod}$* retina sections suggest PRKAA2 and IMPDH reside in the same cellular compartment. PRKAA2 (green) and IMPDH (red) aggregate in the photoreceptor inner segment layer (PIS), but may have slightly more co-localization (orange) only in *Prkaa2$^{-Rhod/-Rhod}$* compared to *Prkaa2$^{F/F}$*. Scale bars represent 40 μm.

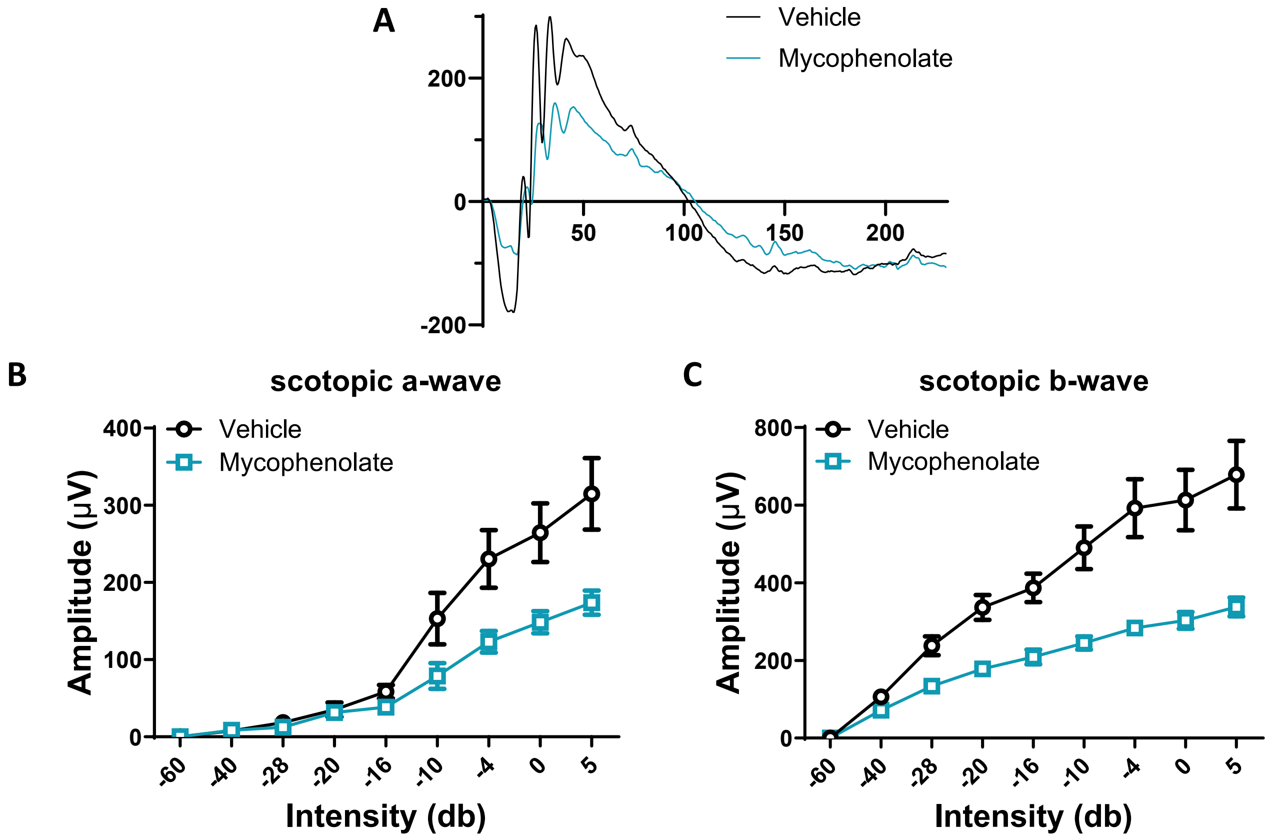

Supplementary Figure 6. Mycophenolate injection does not improve visual function in wild-type mice. (A) A representative ERG trace of mycophenolate and vehicle injected eyes show mycophenolate does not improve visual function as seen in *Prkaa2*$^{-Rhod/-Rhod}$. **(B-C)** Quantifications of scotopic a and b waves demonstrate mycophenolate negatively affects visual function, which is the opposite effect seen in *Prkaa2*$^{-Rhod/-Rhod}$ (n=5). Values are mean ± SE.
